# The Prebiotic Effect of Australian Seaweeds on Commensal Bacteria and Short Chain Fatty Acid Production in a Simulated Gut Model

**DOI:** 10.3390/nu14102163

**Published:** 2022-05-23

**Authors:** Emer Shannon, Michael Conlon, Maria Hayes

**Affiliations:** 1 Teagasc Food Biosciences, Ashtown Food Research Centre, Dunsinea Lane, Ashtown, D15 KN3K Dublin, Ireland; maria.hayes@teagasc.ie; 2 The Commonwealth Scientific and Industrial Research Organisation, Health and Biosecurity, Adelaide, SA 5000, Australia; michael.conlon@csiro.au

**Keywords:** seaweed, prebiotics, in vitro gut model, polysaccharides, polyphenols, fibre, short chain fatty acids, functional food, immunometabolism, Shannon diversity index

## Abstract

Diet is known to affect the composition and metabolite production of the human gut microbial community, which in turn is linked with the health and immune status of the host. Whole seaweeds (WH) and their extracts contain prebiotic components such as polysaccharides (PS) and polyphenols (PP). In this study, the Australian seaweeds, *Phyllospora comosa*, *Ecklonia* *radiata*, *Ulva ohnoi*, and their PS and PP extracts were assessed for potential prebiotic activities using an in vitro gut model that included fresh human faecal inoculum. 16S rRNA sequencing post gut simulation treatment revealed that the abundance of several taxa of commensal bacteria within the phylum Firmicutes linked with short chain fatty acid (SCFA) production, and gut and immune function, including the lactic acid producing order Lactobacillales and the chief butyrate-producing genera Faecalibacteria, Roseburia, Blautia, and Butyricicoccus were significantly enhanced by the inclusion of WH, PS and PP extracts. After 24 h fermentation, the abundance of total Firmicutes ranged from 57.35–81.55% in the WH, PS and PP samples, which was significantly greater (*p* ≤ 0.01) than the inulin (INU) polysaccharide control (32.50%) and the epigallocatechingallate (EGCG) polyphenol control (67.13%); with the exception of *P. comosa* PP (57.35%), which was significantly greater than INU only. However, all WH, PS and PP samples also increased the abundance of the phylum Proteobacteria; while the abundance of the phylum Actinobacteria was decreased by WH and PS samples. After 24 h incubation, the total and individual SCFAs present, including butyric, acetic and propionic acids produced by bacteria fermented with *E. radiata* and *U. ohnoi*, were significantly greater than the SCFAs identified in the INU and EGCG controls. Most notably, total SCFAs in the *E. radiata* PS and *U. ohnoi* WH samples were 227.53 and 208.68 µmol/mL, respectively, compared to only 71.05 µmol/mL in INU and 7.76 µmol/mL in the EGCG samples. This study demonstrates that whole seaweeds and their extracts have potential as functional food ingredients to support normal gut and immune function.

## 1. Introduction

Bacteria, archaea, protozoa, fungi, and viruses are the organisms that comprise the human gut microbiota [1]. Approximately 3.8 × 10^13^ bacterial cells live in the intestines and colon of the average human [2] and constitute more than 90% of all gut microorganisms [3]. The predominant bacterial phyla are Firmicutes, Bacteroidetes, Actinobacteria, and Proteobacteria [4]. These phyla contain a combination of indigenous commensal bacteria which are harmless or beneficial to the host, but may become harmful if overabundant, leading to dysbiosis [5,6,7,8]. Commensal bacteria have essential functions within the gut environment such as the synthesis of vitamins K and B, and the catabolism of food components that are indigestible in the stomach [9]. They are also an integral part of the human immune system. Gut bacteria prevent the colonisation of pathogenic bacteria on the mucosal surface by competing for nutrients and attachment sites, by producing antimicrobial products such as bacteriocins [10,11,12], and by reducing intestinal pH via the production of SCFA and lactic acid [13]. In addition, gut bacteria maintain epithelial integrity via regulation of tight junction permeability, preventing pathogens from entering the blood stream [14]. The mucosal tissues that line the human gastrointestinal tract contain more immune-related cells than all secondary lymphatic tissues of the body combined [15,16]. Changes in the composition and ratio of gut bacterial populations and their metabolites have been found to impact the innate and adaptive immune homeostasis of the host [17,18,19,20,21]. For example, studies in germ-free animals have found impaired activity in gut-associated lymphoid tissues, antibody production, mesenteric lymph nodes, and inflammatory response genes that encode type I interferons [22,23,24,25,26]. This impact on immunometabolism is primarily due to host-beneficial SCFA produced via bacterial fermentation of complex dietary polysaccharides, or saccharolysis [27]. Polysaccharides have structural and energy storage functions within seaweed [28]. The majority of seaweed polysaccharides are classed as fibre and include fucoidan, laminarin, and alginate in brown species; porphyran, agar, floridean starch and carrageenans in red; and ulvan in green [29,30]. Dietary fibre is any edible carbohydrate polymer containing three or more monosaccharides that are resistant to gastric digestive enzymes and pass unhydrolysed through the small intestine [31]. Fibre content ranges broadly amongst red, green, and brown seaweeds from 10–67% depending upon the species and season [32]. Acetic, butyric, propionic, valeric, and caproic acids, as well as dihydrogen gas (H_2_), are produced by gut bacteria as waste by-products of fibre metabolism [33]. Butyric acid is the primary energy source for colonocytes [34]. Colonocytes absorb SCFA via passive diffusion and monocarboxylate transporters [35]. SCFAs are then catabolised in the colonic epithelium into lipids or ketones such as acetoacetate or *β*-hydroxybutyrate [36,37]. In the gut epithelium immune cells, SCFAs activate downstream anti-inflammatory signalling pathways by acting as ligands for the G-protein coupled receptors GPR41, GPR43, and GPR109A [38]. These receptors help to maintain immune homeostasis in the gut by increasing regulatory T cell lymphocyte proliferation [39]. 

Mediators of immune response have been stimulated by seaweed fibre in some previous in vivo studies [40]. In a 57-day feeding trial with weaner piglets, Hui et al. [41] found that the addition of a 2.5% *Ascophyllum nodosum*, *Saccharina latissima*, and rapeseed blend to the animals’ feed relieved gut lymphocyte infiltration, improved the colon mucosa barrier, and positively altered the gut microbiota composition. In a porcine intestinal epithelial cell model, Berri et al. [42] reported that the green seaweed polysaccharide ulvan, from *Ulva armoricana*, increased the expression of cytokines including tumour necrosis factor-*α*, transforming growth factor-*β*, several interleukins, peroxisome proliferator activated receptor-*γ*, toll-like receptor-2, and the chemokine CCL20. In a human trial, Gueven et al. [43] measured an increase in the expression of genes related to immunity such as mitogen-activated protein kinase in blood samples after a single oral ingestion of fucoidan. Other human dietary studies with seaweed fibres did not measure immune response directly, but did have a eubiotic effect by increasing the abundance and diversity of commensal bacteria and their metabolites. Terada et al. [44] found after two weeks of alginate supplementation in eight subjects that levels of Bifidobacteria significantly increased, while Enterobacteriaceae decreased. Acetic and propionic acids also increased, while faecal *p*-cresol, phenol, and indole sulphide, were significantly reduced. Indoles, *p*-cresol, and phenol are produced by some bacteria during fermentation of amino acids in the gut and have been linked with increased risk of cancer and immune disorder [45,46]. Animal dietary studies have shown that fibre-rich seaweed supplementation increases the abundance of beneficial bacteria and SCFA production, while also reducing pathogenic bacterial species [47,48]. For example, supplementation with fucoidan and laminarin from brown seaweeds, and a polysaccharide from red seaweed increased bacterial abundance and SCFA production in pigs, mice, and rats [49,50,51,52].

Seaweeds are also rich in polyphenolic compounds. The polyphenolic content of seaweeds ranges from 1–5% in red and green species, and up to 20% in brown [53,54,55]. In vitro studies using human intestinal microbiota have shown that plant polyphenols can exert positive effects on the balance of intestinal bacteria that are known to influence gut health [56]. For example, Parkar et al. [57] reported an increase in Bifidobacteria and an increase in the ratio of Firmicutes to Bacteroidetes, as well as increased SCFA production with the addition of plant flavonoids such as rutin and quercetin to an in vitro model of human intestinal bacteria. In vivo, polyphenol extracts from plants have also shown positive effects on gut bacteria in human [58] and animal studies [59]. Seaweed polyphenols and their interaction with the gut microbiome have been less studied, but some in vitro and in vivo animal trials have shown beneficial effects. Phlorotannins from *E. radiata* exerted a prebiotic effect in vitro on populations of commensal bacteria such as Bacteroidetes, *Clostridium coccoides*, and *Faecalibacterium prausnitzii* compared to an inulin control in a study by Charoensiddhi et al. [60]. *F. prausnitzii* are major butyric acid producers [61], while commensal *C. coccoides* have been found to play a role in immune homeostasis by inducing the production of T_reg_-cells in murine colonic tissue [62,63]. In a trial with diabetic rats, Yuan et al. [64] reported enhanced gut bacterial diversity after 4 weeks supplementation with a *Lessonia trabeculata* extract of phlorotannins, phenolic acids, and gallocatechins. Compared to the control animals, there was a greater abundance of Bacteroidetes, a greater Firmicutes:Bacteroidetes ratio, less Proteobacteria, and a 61% increase in SCFA production. Similarly, Lin et al. [65] fed a polyphenol mix of luteolin, regiolone, and neoeriocitrin from the green seaweed *Enteromorpha prolifera* to diabetic mice for one month and saw a significant increase in the abundance of the beneficial genera Akkermansia, Alistipes, and Turicibacter. Dietary approaches to enhance the abundance of host-beneficial gut bacterial in human studies have shown promise in recent years with prebiotic plant polysaccharide fibres such as inulin [66,67,68] and polyphenols [58,69,70].

The aim of this study was to evaluate the prebiotic effect of whole seaweeds and their polysaccharide and polyphenol extracts on human faecal gut bacteria using an in vitro model. Extracts were prepared using food-grade solvents and enzymes, dried, and subjected to in vitro gastrointestinal enzymatic digestion, followed by anaerobic colonic bacterial fermentation. After 24 h, the total and individual SCFAs produced by gut bacteria in the model were significantly enhanced by five of the nine seaweed samples compared to INU, and enhanced by all nine samples compared to EGCG. The abundance of many commensal bacteria increased significantly, including Lactobacillales, Faecalibacteria, Roseburia, Blautia, Bifidobacteria, Streptococci, Butyricicocci, Eubacteriaceae, and Barnesiella. The Shannon Diversity index of species was significantly greater in all seaweed-fermented samples compared to INU and EGCG controls after 24 h. 

Clinical trials are required to confirm the observed in vitro effects; however, this study indicates that consuming whole seaweeds and their polysaccharide and polyphenol extracts may have potential prebiotic bioactivities for use as functional foods and supplements.

## 2. Materials and Methods

### 2.1. Chemicals

All chemicals used in this study were analytical or HPLC grade and sourced from Sigma-Aldrich (Bayswater, Melbourne, VIC, Australia) unless otherwise indicated. Water used for all experiments was ultrapure (Milli-Q^®^ IQ 7003/05/10/15, Burlington, MA, USA).

### 2.2. Seaweed Biomass

Seaweeds were harvested in Australia in December 2020. *U. ohnoi* (Chlorophyta) in Townsville, Queensland; and *E. radiata* and *P. comosa* (Phaeophyceae) in Bermagui, New South Wales. Seaweeds were freeze-dried (72 h, −20 °C, 0.01 atm, Labconco FreeZone 7670021, Kansas City, MO, USA), cold milled (Foss CT293 Cyclotec, Hilleroed, Denmark), then passed through a 0.20 mm sieve and stored in vacuum sealed bags at −80 °C. Milled, dried seaweed was used for all extractions and experiments. After solvent extraction, all extracts were freeze-dried, protected from light and stored in sealed, dry tubes at −80 °C.

### 2.3. Proximate Analysis

The moisture content of dried seaweeds was determined gravimetrically using ISO Method 6496:1999 [71] at 105 °C until a constant mass was reached. Ash content was determined using a muffle furnace (550 °C, 7 h, Thermolyne F-A1730, Dubuque, IA, USA) according to ISO method 2171:2007 [72]. Essential mineral trace elements were quantified by the National Measurement Institute, Melbourne, Australia using inductively coupled plasma-mass-spectrometry and inductively coupled plasma atomic emission spectroscopy according to the AOAC Official Methods 986.15 [73] and 974.14 [74], and USEPA Methods 6010 [75] and 6020 [76]. Protein content was determined using a nitrogen analyser (Elementar Rapid MAX N Exceed, Langenselbold, Hesse, Germany) based on the Dumas combustion principle according to the AOAC Official Method 992.23 [77]. The nitrogen-to-protein conversion factors used for seaweeds were: *P. comosa* and *E. radiata*: 4.17, and *U. ohnoi*: 4.24 according to Biancarosa et al. [78]. Total lipids were quantified according to the Bligh and Dyer [79] chloroform-methanol-water method. Total polysaccharides were quantified using the Dubois phenol-sulphuric acid method [80]. Determination of total, soluble, and insoluble dietary fibre was carried out according to the enzymatic-gravimetric AOAC Official Method 991.43-1994 [81] using a dietary fibre analyser (ANKOM, Macedon, New York, NY, USA). Total neutral non-starch polysaccharides were quantified by gas chromatography according to the AOAC Official Method 994.13-1999 [82] for neutral sugar residues. Total phenolic content was determined using the AOAC Official Method 2017.13: Folin and Ciocalteu colorimetric method [83] and compared to gallic acid standards for *U. ohnoi* polyphenols, or phloroglucinol standards for *P. comosa* and *E. radiata* phlorotannins. 

#### Oxygen Radical Absorbance Capacity

Oxygen radical absorbance capacity of WH seaweeds and PP extracts was measured using the AOAC Official Method 2012.23. Total antioxidant activity oxygen radical absorbance capacity was determined using fluorescein as the fluorescence probe [84] and results compared to a series of Trolox standards.

### 2.4. Polysaccharide Extraction

Crude polysaccharides were extracted according to the method of Dore et al. [85]. Pigments and lipids were removed from dried seaweed (10 g) by incubation with acetone and stirring at 200× *g* rpm for 3 h at room temperature. Acetone was removed by centrifugation (5000× *g*, 30 min, SIGMA Model 4-5L, Darmstadt, Germany). The pellet was allowed to dry under a fume hood for 1 h and then suspended in sodium chloride (0.25 M, 100 mL) in capped Duran flasks and adjusted to pH 8.0 (Radiometer PHM93 pH meter, Copenhagen, Denmark) with sodium hydroxide (0.1 M). The bottles were placed in a shaking-waterbath (Thermoline Scientific TSBT-21, Wetherill Park, NSW, Australia) at 60 °C, 200 rpm for 30 min. Subtilisin A protease (10 mg) was added to initiate proteolytic digestion (60 °C, 200 rpm, 24 h) to release polysaccharides from the algal cell protein–polysaccharide complex. The enzyme was deactivated by heating (95 °C, 10 min). The flask contents were allowed to cool to room temperature, then filtered through clean muslin cloth. The filtrate was precipitated with an equal volume of ice-cold acetone on a stirplate (4 °C, 200× *g* rpm, 30 min). Precipitated polysaccharides were collected by centrifugation (10,000× *g*, 30 min) and subsequently freeze dried.

### 2.5. Polyphenol Extraction

#### 2.5.1. Phlorotannins from Brown Seaweeds

Crude phlorotannins were extracted from *P. comosa* and *E. radiata* according to the method of Lopes et al. [86] as described previously.

#### 2.5.2. Polyphenols from Red and Green Seaweeds

Crude polyphenols were extracted from *U. ohnoi* according to the method by Farvin and Jacobsen [87]. Briefly, dried seaweed (5 g) was stirred overnight (200× *g* rpm) with ethanol:water (96:4 *v*/*v*, 50 mL) at room temperature, then centrifuged (2800× *g*, 10 min). The supernatant was retained. The pellet was re-extracted with ethanol:water three times. The pooled supernatants were transferred to a rotary evaporator to remove the ethanol and subsequently freeze-dried. 

### 2.6. Simulated Gastric Digestion

Dried whole seaweeds, polysaccharide extracts, and polyphenol extracts were subjected to simulated gastric digestion according to the in vitro method of Bird et al. [88]. Dried whole seaweed or extract (5 g) was suspended in a porcine pepsin (2000 U/mL) solution (20 mL, pH 2.0) in screwtop containers and incubated in a shaker-waterbath (37 °C, 200× *g* rpm, 30 min). The pH was adjusted to 6.0 with the addition of sodium hydroxide (0.2 M, 20 mL) and acetate buffer (0.2 M, pH 6.0, 80 mL) containing calcium chloride (4.0 mM) and magnesium chloride (0.49 mM). Porcine pancreatin (100 U/mL protease, 60 U/mL lipase, 200 U/mL amylase) was added (20 mL). Amyloglucosidase (30 U/mL, from *Aspergillus niger*) was then added (20 mL) and the container was incubated in a shaker-waterbath (37 °C, 200× *g* rpm, 12 h). The content of each container was poured into pure ethanol (640 mL, 1 h, room temp.) to precipitate the undigested matter. The ethanol was removed by centrifugation (10,000× *g*, 30 min) and disgarded. The pellet was washed with ethanol:water (80:20 *v*/*v*, 200 mL) and centrifuged. The pellet was then washed with acetone (200 mL), centrifuged, freeze dried, and weighed. The digestibility of whole seaweed or extracts was calculated by subtracting the dried, digested pellet mass from the original mass and expressing the mass lost as a percentage of the original.

### 2.7. Simulated Anaerobic Digestion

Preparation of basal fermentation medium, fresh faecal inoculum, and anaerobic fermentation was carried out according to the method of Zhou et al. [89] with minor modifications.

#### 2.7.1. Preparation of Basal Fermentation Medium

To 800 mL ultrapure water was added peptone water (2 mL), yeast extract (2 g), sodium chloride (0.1 g), potassium phosphite (0.04 g), potassium phosphate monobasic (0.04 g), magnesium sulphate (0.01 g), calcium chloride (0.01 g), sodium bicarbonate (2 g), Tween 80 (2 mL), hemin (0.05 g) dissolved in sodium hydroxide (1 M, 1 mL), vitamin K (10 µL), L-cysteine-HCl (0.5 g), bile salts (0.5 g), and resazurin (4 mL, 0.025 g/mL). The volume was brought to one litre and the media was autoclaved (121 °C, 15 min), then transferred to an anaerobic chamber (The Clean Spot, Bactron IV Anaerobic Chamber, Sheldon Manufacturing Inc., Cornelius, NC, USA) for equilibriation overnight.

#### 2.7.2. Fresh Faecal Inoculum

Fresh faecal samples were collected and pooled from three individual healthy human volunteers who were not on any dietary restrictions and had not taken antibiotics for at least 3 months prior to donating. Informed consent was obtained from all subjects involved in the study. Faecal samples were transferred to an anaerobic chamber, and large food particles were removed. An equal mass of faeces from each donor was combined and diluted to 10% (*w*/*v*) with sterile anaerobic phosphate-buffered saline (0.01 M, pH 7.2) and used as the fermentation starter. The slurry was homogenised and constantly stirred during inoculation into each fermentation tube.

#### 2.7.3. Anaerobic Fermentation

Anaerobic fermentation was used to assess the effect of seaweed substrates and controls on the composition of gut bacteria and their SCFA production. Sterile 15 mL polypropylene screwtop tubes were used in triplicate for each substrate, with an aditional sacrifice tube for each set to measure the volume of orthophosphoric acid (0.1 M) required to adjust the pH to 6.8. To each tube was added 100 mg dried whole seaweed, polysaccharide or polyphenol extract, or control substrate. Cellulose was used as a negative control. Inulin and epigallocatechin gallate were used as positive polysaccharide and polyphenol controls, respectively. For the blank, no substrate was added. Sterile basal fermentation medium (9 mL) was added to each tube followed by faecal inoculum slurry (1 mL). The pH of each sacrifice tube was measured and the required volume of orthophosphoric acid to obtain pH 6.8 was noted and added to each corresponding set of samples, controls, and blanks. The tubes were capped, vortexed, and incubated anaerobically (37 °C, 80× *g* rpm, 24 h). After fermentation, tubes were vortexed, centrifuged (500× *g*, 5 min), and aliquots of 1 mL transferred to clean Corning tubes, which were stored at −80 °C for microbial sequencing and SCFA quantification.

### 2.8. Short Chain Fatty Acid Quantification

SCFAs in faecal samples were identified and quantified using gas chromatography according to the method of Watson et al. [90]. To each 1 mL of faecal sample, a heptanoic acid internal standard (1.68 mM, 3 mL) was added. Tubes were vortexed and centrifuged (2095× *g*, 5 min, 4 °C). Supernatants were transferred to fresh microcentrifuge tubes and centrifuged (15, 400× *g*, 5 min, 4 °C). An aliquot of supernatant (300 µL) was acidified with 10% phosphoric acid (10 µL) and filtered (Whatman PTFE 0.45 µm Mini-UniPrep tube). Filtrates were analysed on an Agilent 6890 gas chromatograph (Agilent Technologies, Santa Clara, CA, USA) equipped with a flame ionisation detector and a capillary column (Zebron ZB-FFAP, 30 m × 0.53 mm × 1.0 µm, Phenomenex, Lane Cove, NSW, Australia) and compared to standards for acetic, butyric, propionic, iso-butyric, valeric, iso-valeric, and caproic acids. 

### 2.9. Bacterial 16S rRNA Sequencing

The percentage relative abundance of bacteria within each taxa from phylum to species in the fermented faecal samples after 24 h incubation were determined by 16S rRNA gene sequencing using the Roche 454 platform at the Australian Genome Research Facility (AGRF, University of Queensland, Brisbane, QLD, Australia). The V3-V4 region of bacterial samples was sequenced on an Illumina MiSeq platform. The bioinformatic analysis involved demultiplexing, quality control, Amplicon Sequence Variant (ASV) calling, and taxonomic classification. Primer details: Target 341F. Forward primer CCTAYGGGRBGCASCAG, reverse primer GGACTACNNGGGTATCTAAT, amplicon sequencing read length 300 bp.

### 2.10. Bioinformatics Methods

Diversity profiling analyses were performed using Quantitative Insights Into Microbial Ecology software (QIIME 2 version 2019.7, Caporaso Lab Pathogen and Microbiome Institute, Northern Arizona University, Flagstaff, AZ, USA) according to the method of Bolyen et al. [91]. The demultiplexed raw reads were primer trimmed and quality filtered using the QIIME 2 cut-adapt plugin followed by denoising with DADA2 according to Callahan et al. [92] (via q2-dada2). Taxonomy was assigned to ASVs using the Bokulich et al. [93] q2-feature-classifier classify-sklearn naive Bayes fitted taxonomy classifier in QIIME 2. Shannon diversity and richness were calculated for each sample by dividing observed operational taxonomic units (OTUs) by ASVs using QIIME2 (v 2019.7). The diversity metrics and the taxonomic composition matrix for each rank were imported into R (version 4.0.2) (R Core Team 2013. R Foundation for Statistical Computing, Vienna, Austria). Absolute counts were converted into total-sum scaling (TSS) and square root transformed. Linear, mixed-effect regression was applied to identify differentially different taxa and diversity indices between groups using the linear mixed-effects models (lmer) function in the R package lme4 according to Kuznetsova et al. [94]. The model contained each taxon as the dependent variable and the interaction of time-point and treatment as fixed effect and plant (seaweed) id as fixed effect: Diversity~Treatment * time.point + (1|plant.id) Taxon~Treatment * time.point + (1|plant.id). Pairwise comparison of least square means was performed using the function lsmeans in R with Tukey’s post-hoc test. ANOVA was applied to identify taxonomic differences between different treatments and time points. For the model, an interaction of treatments and time points was used. Tukey’s test was used for multiple comparisons. *p*-values were reported for the comparison between the different treatments at 24 h. *p*-values of ≤0.05 were considered significant.

### 2.11. Statistical Analysis

All analyses were conducted in triplicate and expressed as means ± standard deviation (SD). One-way ANOVA and Tukey’s HSD post-hoc test (GraphPad Prism 9.1.0, San Diego, CA, USA) was used to assess statistically significant differences between means at the 95% confidence interval.

## 3. Results

### 3.1. Compositional Content

The proximate composition of whole seaweeds and their polysaccharide extracts are presented in Table 1 and total phenolic contents in Table 2. The soluble and insoluble fibre content of all three seaweeds increased up to two-fold after polysaccharide extraction. The soluble and insoluble neutral non-starch polysaccharides (NNSP) of WH seaweeds and their PS extracts are detailed in Table 3. In WH seaweeds, total soluble NNSP ranged from 0.89% in *U. ohnoi* to 6.00% in *P. comosa*, while total insoluble NNSP ranged from 2.60% in *E. radiata* WH to 6.99% in *U. ohnoi* WH. There was more than a 100% increase in both soluble and insoluble NNSP in the polysaccharide extracts compared to WH seaweeds. Total soluble NNSP in PS extracts ranged from 1.66% in *U. ohnoi* PS to 10.17% in *P. comosa* PS. Total insoluble NNSP content of PS extracts ranged from 4.72% in *E. radiata* PS to 10.91% in *P. comosa* PS.

The individual trace mineral contents of WH seaweeds are presented in Table 4. Essential minerals were present in all seaweeds, particularly calcium (2.79–13.00 mg/g), magnesium (5.76–32.00 mg/g), potassium (25.00–78.00 mg/g) and iodine (0.0029–3.40 mg/g). Iodine levels ranged from the lowest in *U. ohnoi* (0.0029 mg/g) to 3.40 mg/g in *E. radiata*. 

The oxygen radical absorbance capacity of WH seaweeds and their PP extracts are presented in Table 5 and expressed as ORAC units (µmol Trolox equivalent (TE)/g). An approximate three-fold increase in ORAC capacity was exerted by the PP extracts compared to the WH seaweeds. The ORAC values of the three seaweeds correlate positively with the total polyphenolic contents observed for each seaweed and extract in Table 1. Values ranged from 17.5 ± 1.05 to 111.0 ± 2.46 µmol TE/g in WH seaweeds, and from 59.1 ± 1.96 to 345.4 ± 6.87 µmol TE/g in PP extracts. The seaweed with the highest ORAC value, *E. radiata* (WH 111.0 ± 2.46 µmol TE/g and PP 345.4 ± 6.87 µmol TE/g), also had the highest total polyphenolic content (WH 0.55 ± 0.03 mg PE/g and PP 3.48 ± 0.27 mg PE/g). This was followed by *P. comosa*, then *U. ohnoi*. 

### 3.2. Simulated Gastric Digestion

The percentage of WH seaweed digested after simulated gastric digestion with pepsin, pancreatin, and amyloglucosidase is detailed in Table 6. Digestibility ranged from 20.28 ± 0.93% in *P. comosa* WH to 41.98 ± 1.84% *E. radiata* WH. Gastric digestibility decreased significantly after polysaccharide extraction, ranging from 8.38% ± 1.53 in *U. ohnoi* PS to 11.77 ± 1.94% in *E. radiata* PS.

### 3.3. Short Chain Fatty Acid Production

Table 7 and Supplementary Figure S1 show the total and individual SCFA concentration produced by bacteria in the in vitro model after 24 h incubation with WH seaweed, PS or PP extracts, or controls. Compared to the INU polysaccharide control (71.05 ± 1.08 µmol/mL), a highly significant increase in total SCFA production occurred in the seaweed-containing ferments, most notably in *E. radiata* PS (227.53 ± 5.39 µmol/mL), *U. ohnoi* WH (208.68 ± 19.08 µmol/mL), *E. radiata* PP (183.73 ± 20.06 µmol/mL), and *U. ohnoi* PS (182.91 ± 3.47 µmol/mL). The four exceptions were *P. comosa* WH, PS and PP, and *E. radiata* WH, which ranged from only 37.38 ± 0.74 µmol/mL to 61.20 ± 1.02 µmol/mL of the total SCFAs. However, these four samples still produced more SCFA than the EGCG control (7.76 ± 0.09 µmol/mL), cellulose (35.55 ± 1.45 µmol/mL), and the blank (33.53 ± 2.03 µmol/mL). 

Acetic acid was the most prevalent individual SCFA present in all samples. This was followed by approximately equal increases in butyric and propionic acid, then valeric, iso-valeric, iso-butyric, and caproic acid. Iso-butyric, iso-valeric, and caproic acids were absent in INU and EGCG after 24 h incubation, but were produced by bacteria when fermented with all seaweed extracts. 

### 3.4. Bacterial Abundance

**Figure 1:**
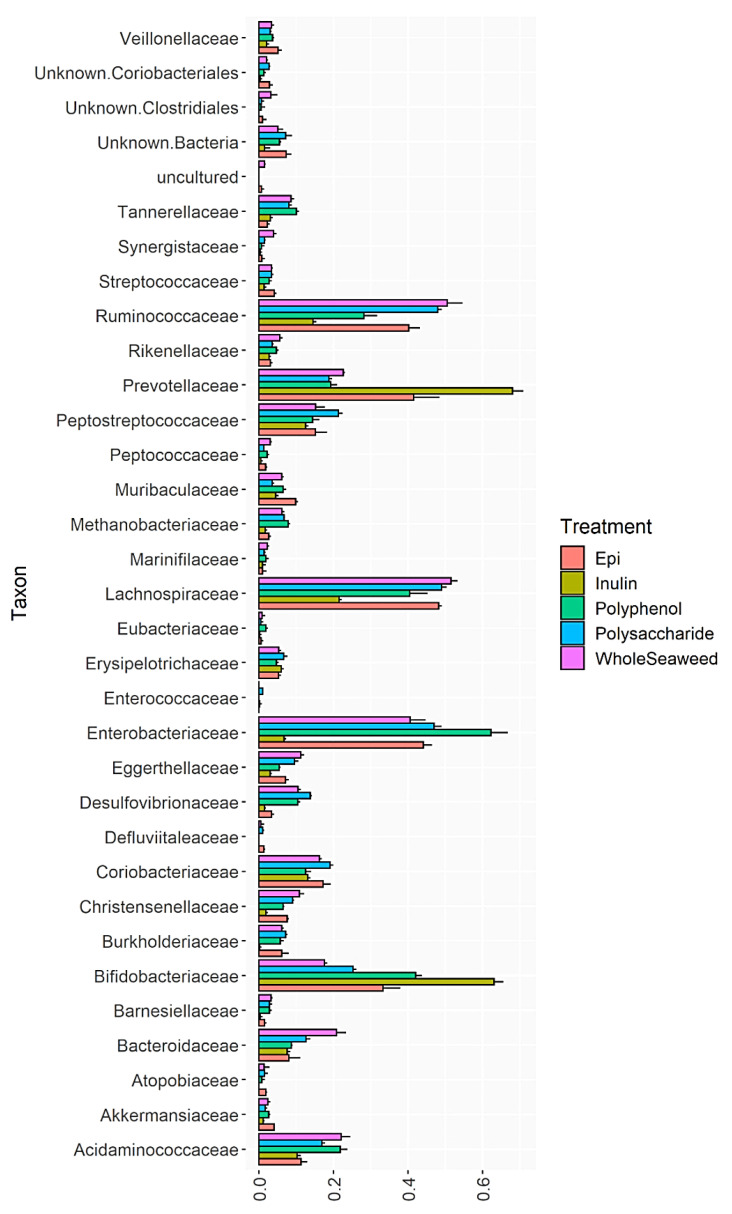
Relative abundance of bacterial families after 24 h fermentation with *P. comosa* WH, PS, PP, INU or EGCG.

**Figure 2:**
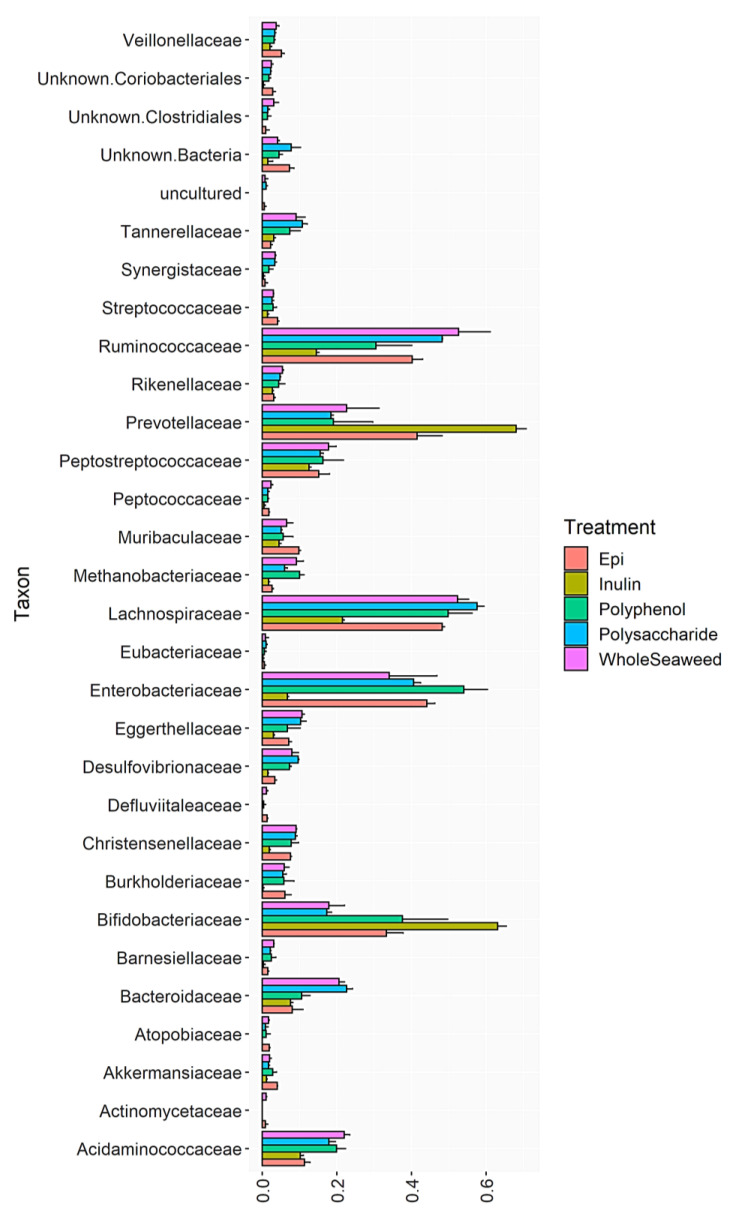
Relative abundance of bacterial families after 24 h fermentation with *E. radiata* WH, PS, PP, INU or EGCG.

**Figure 3:**
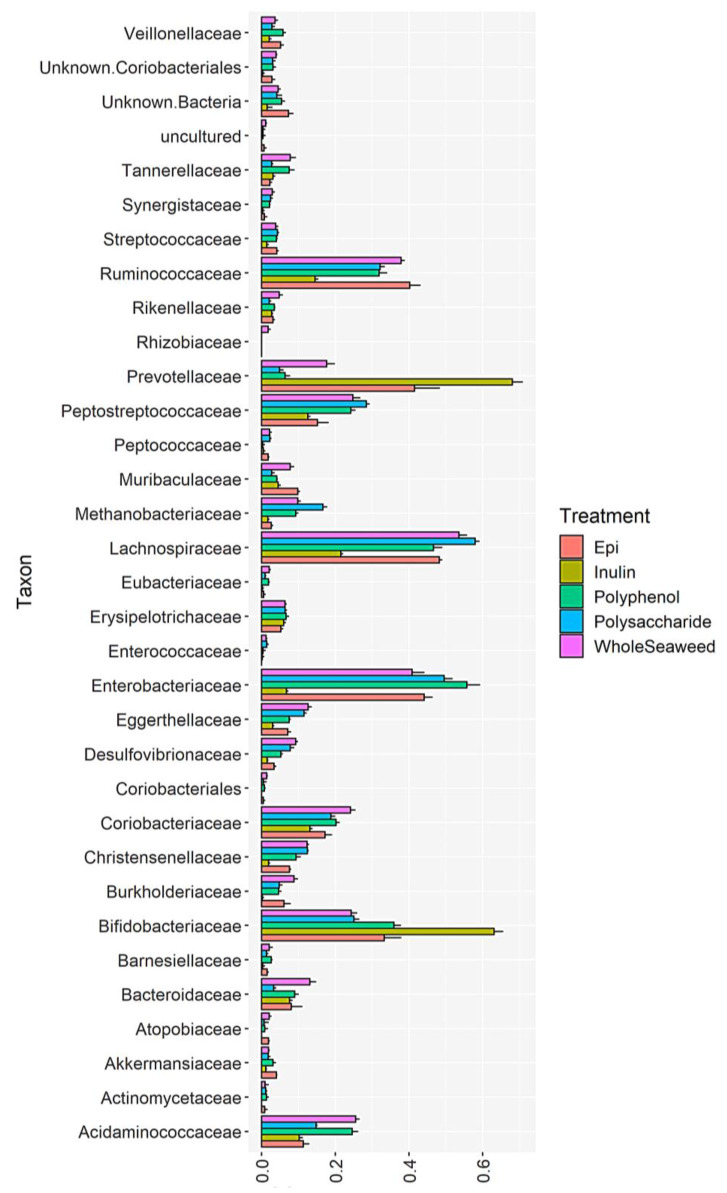
Relative abundance of bacterial families after 24 h fermentation with *U. ohnoi* WH, PS, PP, INU or EGCG.

Table 8 details the relative abundance of bacterial groups at 24 h post-fermentation that were significantly enhanced or decreased by WH, PS, or PP seaweed extracts compared to the INU or EGCG controls. Figure 1, Figure 2 and Figure 3 and Supplementary Figures S2–S4 show the impact of different seaweed substrates on the relative abundance of bacteria at the family level within the gut microbiota after 24 h. Only families with a relative abundance of 0.01% or more were included.

The total abundance of the phylum Firmicutes was significantly enhanced by all WH, PS and PP extracts (ranging from 57.35–81.55%) compared to INU (32.50%) (Supplementary Figures S5–S8) and EGCG (67.13%); with the exception of *P. comosa* PP (57.35%), which was significantly greater than INU only (Supplementary Figure S9). Within the phylum Firmicutes, the abundance of the lactic acid producing order Lactobacillales, particularly the genus Streptococcus, increased significantly compared to INU (1.44% abundance) in all WH, PS and PP extracts (Supplementary Figures S10 and S11), but was not enhanced compared to EGCG (4.07%). The abundance of the family Eubacteriaceae (Supplementary Figure S12), was enhanced by all WH, PS, and PP extracts compared to INU and EGCG; with the exception of the *E. radiata* PP ferment which was slightly more abundant than EGCG, but not significantly. Notably, the beneficial species *Eubacterium halii* was enhanced significantly by all WH, PS, and PP extracts compared to INU. However, compared to EGCG, only the *E. radiata* PP sample increased *E. halii* abundance (Supplementary Figure S13).

The enhanced Firmicutes genera were Faecalibacteria (Supplementary Figure S14), Butyricicoccus (Supplementary Figure S15), Roseburia (Supplementary Figure S16), and Blautia (Supplementary Figure S17). The same pattern was observed in these genera where abundance was significantly greater in all WH, PS, and PP extracts compared to INU but not compared to EGCG. Similar increases in abundance compared to INU were induced by WH and PS in the genus Akkermansia (phylum Verrucomicrobiota) (Supplementary Figure S18), coupled with slight decreases by PP. However, none of the PP decreases were significant apart from *P. comosa* PP, which reduced Akkermansia to 2.62% abundance (*p* = 0.0058) compared to 4.00% in the EGCG control. 

Increases were observed in phylum Proteobacteria and decreases in phylum Actinobacteria, also known as Actinomycetes. All nine seaweed substrates induced significant increases in Proteobacteria compared to INU and EGCG. All WH and PS seaweed substrates reduced the abundance of Actinobacteria compared to INU. There were, however, slight increases in Actinobacteria abundance with the *P. comosa*, *E. radiata*, and *U. ohnoi* polyphenol extracts compared to EGCG, but they were not significant. There was one exception within the phylum Actinobacteria. The order Bifidobacteria was enhanced only by *P. comosa* PP (42.01%) compared to EGCG (33.30%) (*p* = 0.00648) (Supplementary Figure S19). Lastly, all PS and WH seaweed substrates significantly enhanced the abundance of an unclassified phylum termed ‘Bacteria’ compared to INU (Supplementary Figure S20), while the abundance of another group, the ‘Human Gut Metagenome’ (Supplementary Figure S21), was enhanced by all seaweed ferments (WH, PS, and PP) versus INU.

Table 8 shows the Shannon Diversity Index of total species. This is calculated from the number of operational taxonomic units observed within each taxon. Species diversity was significantly greater than INU (3.38) in all WH, PS, and PP seaweed ferments (ranging from 4.79 to 6.46) (Supplementary Figure S22). However, no seaweed extracts had significantly greater diversity than the EGCG control (6.43).

**Table 8:** Relative abundance (% of total abundance) of selected bacterial groups at 24 h that were significantly enhanced or decreased by WH, PS, or PP seaweed extracts compared to the INU or EGCG controls.

	Phylum					Order	Genus					Species	
	Gram +	Gram -	Gram +	Gram -	/	Gram +	Gram +	Gram -	Gram +	Gram -	Gram +	Gram +	
Substrate	Firmicutes	Bacteroidetes	Actinobacteria	Proteobacteria	Unclassified Bacterial Phylum	Lactobacillales	Faecalibacteria	Akkermansia	Roseburia	Barnesiella	Butyricicoccus	*B. hydrogenotrophica*	Shannon Diversity Index
Inulin	32.50	68.71	64.48	6.91	1.47	1.44	10.63	1.15	3.08	0.26	3.04	8.67 × 10^−17^	3.38
EGCG	67.13	43.16	38.32	44.67	7.24	4.07	24.03	4.00	18.14	1.52	6.72	0.41	6.43
*P. comosa* WH	↑† 79.42 (*p* = 5.95 × 10^−10^)	↓† 33.20 (*p* = 4.97 × 10^−7^)	↓† 26.47 (*p* = 8.50 × 10^−9^)	↑† 42.32 (*p* = 6.79 × 10^−8^)	↑† 5.03 (*p* = 0.0132)	↑† 3.36 (*p* = 9.7 × 10^−4^)	↑† 20.54 (*p* = 2.4 × 10^−6^)	↑† 2.34 ^NS^	↑† 12.27 (*p* = 2.08 × 10^−7^)	↑† 3.23 (*p* = 0.0017)	↑† 7.46 (*p* = 0.0004)	↑† 3.32 (*p* = 0.00087)	↑† 6.46 (*p* = 5.45 × 10^−14^)
*P. comosa* PS	↑† 75.73 (*p* = 2.63 × 10^−3^)	↓† 24.70 (*p* = 1.56 × 10^−7^)	↓† 33.18 (*p* = 2.26 × 10^−8^)	↑† 49.47 (*p* = 4.32 × 10^−11^)	↑† 7.18 (*p* = 0.0030)	↑† 3.50 (*p* = 0.0050)	↑† 13.22 (*p* = 0.0126)	↑† 1.70 ^NS^	↑† 7.06 (*p* = 6.21 × 10^−5^)	↑† 2.76 (*p* = 0.0080)	↑† 6.81 (*p* = 7.70 × 10^−5^)	↓† 4.34 × 10^−17^ ^NS^	↑† 5.77 (*p* = 4.02 × 10^−9^)
*P. comosa* PP	↓§ 57.35 (*p* = 0.0092)	↓§ 24.87 (*p* = 0.0013)	↑§ 44.19 ^NS^	↑§ 63.31 (*p* = 6.66 × 10^−5^)	↓§ 5.53 ^NS^	↓§ 2.68 (*p* = 0.0195)	↓§ 19.06 (*p* = 0.0470)	↓§ 2.62 ^NS^	↓§ 5.51 (*p* = 2.80 × 10^−5^)	↑§ 2.80 (*p* = 0.0340)	↓§ 4.29 (*p* = 0.0126)	↑§ 0.48 ^NS^	↓§ 4.79 (*p* = 4.26 × 10^−6^)
*E. radiata* WH	↑† 81.55 (*p* = 6.97 × 10^−8^)	↓† 33.41 (*p* = 5.94 × 10^−5^)	↓† 25.92 (*p* = 1.57 × 10^−6^)	↑† 35.67 (*p* = 0.0017)	↑† 4.09 (*p* = 0.0137)	↑† 3.03 (*p* = 0.0107)	↑† 22.30 (*p* = 0.0019)	↑† 1.97 ^NS^	↑† 11.17 (*p* = 0.0002)	↑† 3.06 (*p* = 0.0007)	↑† 7.42 (*p* = 0.0005)	↑† 3.27 (*p* = 0.00092)	↑† 6.25 (*p* = 2.84 × 10^−5^)
*E. radiata* PS	↑† 80.44 (*p* = 0.0000)	↓† 31.87 (*p* = 9.01 × 10^−7^)	↓† 25.0 (*p* = 9.46 × 10^−9^)	↑† 42.02 (*p* = 1.37 × 10^−9^)	↑† 7.71 (*p* = 0.0035)	↑† 2.57 (*p* = 0.0496)	↑† 20.91 (*p* = 7.03 × 10^−6^)	↑† 1.70 ^NS^	↑† 12.45 (*p* = 2.11 × 10^−6^)	↑† 2.10 (*p* = 0.0111)	↑† 8.39 (*p* = 5.44 × 10^−5^)	↑† 3.34 (*p* = 7.83 × 10^−5^)	↑† 6.46 (*p* = 7.69 × 10^−14^)
*E. radiata* PP	↓§ 65.53 ^NS^	↓§ 24.53 (*p* = 0.0285)	↑§ 41.31 ^NS^	↑§ 57.81 (*p* = 0.0279)	↓§ 4.45 (*p* = 0.0359)	↓§ 2.92 ^NS^	↓§ 15.13 (*p* = 0.0004)	↓§ 2.82 ^NS^	↓§ 6.76 (*p* = 5.53 × 10^−5^)	↑§ 2.39 (*p* = 0.0464)	↓§ 5.95 ^NS^	↑§ 2.40 (*p* = 0.04372)	↓§ 5.25 (*p* = 0.0008)
*U. ohnoi* WH	↑† 77.73 (*p* = 0.00)	↓† 25.16 (*p* = 1.5 × 10^−6^)	↓† 36.78 (*p* = 9.31 × 10^−8^)	↑† 42.79 (*p* = 1.06 × 10^−8^)	↑† 4.51 (*p* = 5.26 × 10^3^)	↑† 4.01 (*p* = 0.0013)	↑† 17.31 (*p* = 0.0003)	↑† 1.82 ^NS^	↑† 9.57 (*p* = 1.08 × 10^−5^)	↑† 2.01 (*p* = 0.0491)	↑† 8.49 (*p* = 2.35 × 10^−6^)	↑† 4.35 (*p* = 0.00013)	↑† 6.38 (*p* = 3.56 × 10^−13^)
*U. ohnoi* PS	↑† 77.28 (*p* = 0.00)	↓† 0.740 (*p* = 4.14 × 10^−8^)	↓† 33.54 (*p* = 3.20 × 10^−8^)	↑† 50.38 (*p* = 2.30 × 10^−10^)	↑† 4.12 (*p* = 0.0363)	↑† 4.52 (*p* = 4.92 × 10^−5^)	↑† 13.62 (*p* = 0.0313)	↑† 1.77 ^NS^	↑† 8.80 (*p* = 3.82 × 10^−5^)	↑† 1.31 (*p* = 0.0500)	↑† 7.00 (*p* = 0.0003)	↑† 3.32 (*p* = 7.02 × 10^−5^)	↑† 5.78 (*p* = 2.89 × 10^−9^)
*U. ohnoi* PP	↑§ 68.80 ^NS^	↓§ 14.63 (*p* = 6.47 × 10^−5^)	↑§ 42.00 ^NS^	↑§ 56.07 (*p* = 0.0007)	↓§ 5.41 ^NS^	↓§ 4.06 ^NS^	↓§ 21.89 ^NS^	↓§ 3.03 ^NS^	↓§ 6.81 (*p* = 7.40 × 10^−8^)	↑§ 2.64 (*p* = 0.0061)	↑§ 6.92 ^NS^	↓§ 2.32 × 10^−17^ (*p* = 3.26 × 10^−6^)	↓§ 5.22 (*p* = 1.15 × 10^−5^)

† Significantly different than INU; § significantly different than EGCG; ^NS^ no significant increase or decrease vs. INU or EGCG.

The ratio of bacteria within the phyla Firmicutes and Bacteroidetes in seaweed ferments, INU, EGCG or cellulose standards, and the blank at 24 h are shown in Table 9. The F/B ratio in the EGCG control was 0.671/0.436 (1.539). The F/B ratio in the INU control was 0.325/0.687 (0.473), i.e., there was more than double the abundance of Bacteroidetes compared to Firmicutes when inulin was used as a substrate. The reverse was seen in all nine seaweed ferments where Firmicutes were more abundant. F/B ratios ranged from 2.301 in *P. comosa* PP to 10.446 in *U. ohnoi* PS. However, the cellulose negative control and the blank had ratios in the same range (3.228 and 3.484, respectively); therefore, the changes in F/B ratios may not have been due to the effect of seaweed components. Only two seaweed extracts had greater F/B ratios than cellulose and the blank. These were *U. ohnoi* PP (4.712) and PS (10.446). 

## 4. Discussion

Diet impacts the abundance of gut bacteria and their production of SCFAs. This, in turn, affects the immune system as gut bacteria prevent pathogenic microorganisms from colonising the gut lining and entering the blood stream [14], while SCFAs activate anti-inflammatory signalling pathways in the gut epithelium immune cells [38] which increases immune regulatory T cell lymphocyte proliferation [39]. Since the link between the human gut microbiota and overall health has been established, approaches to enhance the abundance of commensal gut bacteria include the consumption of prebiotics (fibre), probiotics (live bacteria capable of reaching the gut), and even faecal microbiome transplants [95]. In this study, three edible, commercially available Australian seaweeds—*P. comosa*, *E. radiata*, and *U. ohnoi*—and their PS or PP extracts were assessed for their potential prebiotic activities using an in vitro gut model with human faecal inoculum. 

The proximate analysis (Table 1) showed that all three seaweeds are a considerable source of soluble and insoluble fibre, protein, minerals, and antioxidant polyphenols. Essential minerals were present in all seaweeds, particularly calcium (2.79–13.00 mg/g), magnesium (3.76–32.00 mg/g), and potassium (14.50–78.00 mg/g). Similar contents have been reported previously in these species [96,97]. The high levels of sodium (23.1–30.2 mg/g) and chloride (47.5–101.0 mg/g) may be due to the salt in residual dried sea water absorbed by the thallus. Iodine levels ranged from the lowest in *U. ohnoi* (0.0029 mg/g) to 3.40 mg/g in *E. radiata*. Iodine can cause concern if ingested above the WHO Tolerable Daily Intake limit of 10 mg/kg body mass/day [98]. However, in order to exceed this threshold, 235.29 g of dried *E. radiata* would need to be consumed by a human with a body mass of 80 kg. Similar ash, protein, fibre, lipid, and phenolic contents have been reported previously for *P. comosa*, *E. radiata*, and *U. ohnoi* harvested in the Australasian region [55,99,100,101,102,103,104]. There was a marked increase (from one and a half to two-fold) in the soluble and insoluble fibre, and total polysaccharide content of all three seaweeds (Table 1) after crude polysaccharide extraction. This indicates the successful removal of protein from the seaweed cell protein–polysaccharide complex during enzymatic hydrolysis. The increased fibre content in the extracts improves their prebiotic potential as fibre is the preferred food of gut bacteria [1,6,12].

The antioxidant capacity of each seaweed and PP extract, expressed as ORAC values (Table 5), correlated directly with the total polyphenolic and phlorotannin contents (Table 1). This pattern suggests that the compounds exerting the most prevalent antioxidant effect were the phlorotannins and polyphenols, not other antioxidants such as selenium or vitamins A and C [105]. The ORAC assay shows the capacity of food components to act as antioxidants by donating electrons to the unpaired electrons in the atomic orbital of free radicals [106] and is considered to be the most relevant to human antioxidant biology with a realistic indication of in vivo activity [107,108]. Both lower & higher ORAC values have previously been reported for *P. comosa* (38.8 to 469.64 μmol TE/g) [109] and *E. radiata* (512.4 to 773.5 μmol TE/g) [103]. No published data are available on the ORAC values for *U. ohnoi*. However, in other antioxidant assays, Sáez et al. [110] reported *U. ohnoi* as having a ferric ion reducing antioxidant power of 8.37 to 16.52 μmol TE/g. The ORAC values in the present study may be slightly lower than some published values due to seasonal variations and the influence of geographic location on polyphenolic levels amongst seaweeds [111,112,113,114].

The neutral non-starch polysaccharide profile of each seaweed (Table 3) shows the individual polysaccharides that comprise the soluble and insoluble fibre content. As expected, rhamnose was found only in *U. ohnoi*, where it forms the backbone of the principle green seaweed polysaccharide, ulvan, along with lower levels of xylose. Soluble galactose occurred at low levels, from 0.02 ± 0.00% in *U. ohnoi* WH to 0.92 ± 0.08% in *P. comosa* PS. Fucose, of which the brown seaweed polysaccharide fucoidan is composed, was found only in *E. radiata* and *P. comosa* as expected. Glucose was highest in the two brown species since one of the chief polysaccharides in Phaeophyceae, laminarin, is composed of repeating glucose units. Ribose was detected only in *U. ohnoi* WH (0.09 ± 0.01%) and *U. ohnoi* PS (0.16 ± 002%). Mannose, which is a derivative of mannuronic acid that forms alginate, was most prevalent in *P. comosa*. Similar NNSP contents have previously been reported in polysaccharide extracts of *E. radiata*, *P. comosa*, and Ulva species [115,116,117]. 

An inverse correlation was observed between the extent of simulated gastric digestion (Table 6) and the corresponding total neutral non-starch polysaccharide content of each seaweed (Table 3). In ascending order of digestion, *P. comosa* WH (20.28 ± 0.93%), *U. ohnoi* WH (26.90 ± 1.07%), and *E. radiata* WH (41.98 ± 1.84%) had decreasing levels of total combined soluble and insoluble NNSPs (12.08 ± 1.04%, 7.88 ± 0.93% and 4.53 ± 0.64%, respectively). This is most likely due to the fact that mammalian gastric enzymes are not capable of degrading NNSPs (fibre), which pass intact through the stomach and are digested by the gut bacteria [118]. Gastric digestibility decreased significantly after polysaccharide extraction from 8.38 ± 1.53% in *U. ohnoi* PS to 11.77 ± 1.94% in *E. radiata*, which correlates with the increased NSPP contents of PP extracts and the prebiotic effect they had on SCFA production and bacterial abundance. 

SCFA production was enhanced by five of the nine seaweed samples (Table 7). After 24 h, total and individual SCFAs, including butyric, acetic, and propionic acids, produced by bacteria fermented with *E. radiata*, and *U. ohnoi* were significantly greater than the inulin (INU) polysaccharide control and the epigallocatechingallate (EGCG) polyphenol control. Most notably, total SCFA in *E. radiata* PS was (227.53 ± 5.39 µmol/mL) and PP (183.73 ± 20.06 µmol/mL); *U. ohnoi* WH (208.68 ± 19.08 µmol/mL), PS (182.91 ± 3.47 µmol/mL), and PP (140.42 ± 2.93 µmol/mL) compared to INU (71.05 ± 1.08 µmol/mL) and EGCG (7.76 ± 0.09 µmol/mL). No significant increase in SCFAs occurred in *P. comosa* WH, PS, or PP ferments or in *E. radiata* WH, which ranged from 37.38 ± 0.74 µmol/mL to 61.20 ± 1.02 µmol/mL total SCFAs. However, these seaweed samples did produce significantly more SCFAs than the EGCG control (7.76 ± 0.09 µmol/mL), cellulose (35.55 ± 1.45 µmol/mL), and the blank (33.53 ± 2.03 µmol/mL). Increases in individual SCFAs were primarily seen in acetic, butyric, propionic, and valeric acids, which were 50.33 ± 0.77 µmol/mL, 7.32 ± 0.06 µmol/mL, 13.00 ± 0.25 µmol/mL, and 0.40 ± 0.01 µmol/mL, respectively, in INU, but rose to 145.03 ± 4.38 µmol/mL, 30.04 ± 0.40 µmol/mL, 31.35 ± 0.25 µmol/mL, and 8.58 ± 0.07 µmol/mL in *E. radiata* PS, for example. In addition, iso-butyric, iso-valeric, and caproic acids were absent in INU and EGCG, but were produced by bacteria (4.40 ± 0.10 µmol/mL, 6.60 ± 0.13 µmol/mL and 1.53 ± 0.06 µmol/mL, respectively) when fermented with *E. radiata* PS. The greater production of butyric acid in the seaweed ferments compared to INU and EGCG correlates with the microbial sequencing results which showed increased abundance of butyric acid producing genera of the phylum Firmicutes and a decrease in acetic and propionic acid producing Bacteroidetes. These highly significant increases in SCFA production show the suitability of seaweeds, particularly polysaccharide extracts and whole seaweed thallus, as substrates for gut bacteria.

All nine seaweed substrates had significant impacts on the abundance of commensal bacteria (Table 8 and Figure 1, Figure 2 and Figure 3). At phylum level after 24 h, Bacteroidetes, Firmicutes, Actinobacteria, and Proteobacteria comprised 98.6% of the total bacterial population. Several orders, genera, and species of commensal bacterial associated with gut health were enhanced compared to the controls. The abundance of genera within the phylum Firmicutes linked to butyric acid production, gut function, and immunity were enhanced by all three WH seaweeds (77.73–81.55%), PS (75.73–80.44%), and PP (57.35–68.80%) extracts compared to INU (32.50%) and EGCG (67.13% abundance), with the exception of *P. comosa* PP (57.35%) which was only significantly greater than INU not EGCG. The abundance of the order Lactobacillales—a probiotic of the phylum Firmicutes—increased significantly compared to INU when fermented with all WH and PS extracts, but decreased with all PP extracts compared to EGCG. The same increases in abundance were induced by WH/PS and decreases by PP in the chief butyrate-producing genera Faecalibacterium (family Ruminococcaceae), Roseburia, and Butyricicoccus (both family Lachnospiraceae) compared to the INU and EGCG controls. Roseburia species have been shown to regulate gut barrier homeostasis and cytokine production in vivo by secreting an immune activator called flagellin [119]. Butyricicoccus is a prevalent butyric acid producer and has been used successfully as an encapsulated probiotic in human clinical trials [120]. The inhibition of growth by seaweed polyphenol extracts in the genera Faecalibacteria and Roseburia may be due to the unsuitability of polyphenols as substrates for these bacteria which require polysaccharides for growth. Some in vitro [121] and in vivo studies on the effect of plant polyphenols on gut bacteria found that dietary polyphenols inhibited the growth of Firmicutes [59]. Aside from their unsuitability as a food for some bacteria, some polyphenols from terrestrial [122] and marine [123] plants can also have antimicrobial effects. The phloroglucinols in brown seaweed phlorotannins and the phenolic acids in green seaweed polyphenols contain hydroxyl groups which can bind with the amino groups of proteins [124,125]. This induces cell lysis in bacterial proteins [126]. For example, a recent study by Ford et al. [127] found that the addition of phlorotannins from *A. nodosum* and *F. serratus* to dried pig feed inhibited three foodborne pathogens (*Salmonella agona*, *Escherichia coli* O157, and *Streptococcus suis*) without damaging intestinal cells. The minimum inhibitory concentrations of the phlorotannins against the bacteria ranged from 0.78 to 1.56 mg/mL (*A. nodosum*) and 3.13 mg/mL for all three (*F. serratus*). Since the polyphenol and phlorotannin extracts used in the present study ranged from 2.46 ± 0.21 mg GAE/g to 4.33 ± 0.15 mg PE/g, this may have contributed to the reduced abundance of Firmicutes.

Another important butyric acid-producing species of the Firmicutes, *Intestinimonas butyriciproducens* (class Clostridia, order Clostridiales), increased in abundance (1.69%) compared to INU (1.00 × 10^−16^%) (*p* = 10.45 × 10^−6^) when fermented with *U. ohnoi* WH but not in any other sample (Supplementary Figure S23). *I. butyriciproducens* has the unusual ability to convert glycated, non-bioavailable lysines such as Nε-fructosyllysine into beneficial butyrate in the gut [128]. Some amino acids such as lysine react with reducing sugars, particularly lactose, in the digestive tract and become unavailable to the host, but can be metabolised by *I. butyriciproducens* [129].

The abundance of the genus Barnesiella (order Bacteroidales, phylum Bacteroidota or Sphingobacteria) was enhanced in all seaweed extracts (ranging from 1.31% in *U. ohnoi* PS to 3.23% in *P. comosa* WH) compared to INU (0.26%) and EGCG (1.52%) (Supplementary Figure S24). Barnesiella species have been used in vivo to positively regulate the composition of the microbiota by restricting the growth of vancomycin-resistant Enterococcus [130] and to enhance the anti-cancer immunomodulatory activity of cyclophosphamide [131]. Other less populous genera also grew more abundantly in some seaweed extracts compared to the controls, including Blautia and Eubacteria. Although these genera constitute less than 10% of total gut bacterial abundance, they have pivotal functions within the gut environment and are considered beneficial to the host due to their saccharolytic and SCFA-producing abilities [132,133,134]. In addition to producing butyric acid, Eubacteria, particularly the species *Eubacterium hallii*, *Eubacterium ramulus*, and *Eubacterium ventrosum*, influence immunomodulation and suppression of inflammation in the gut, as well as the transformation of cholesterol and bile acid [135,136]. The genus Blautia (family Lachnospiraceae), specifically *Blautia hydrogenotrophica*, increased in abundance compared to INU and EGCG in six of the nine seaweed ferments. The three exceptions were *P. comosa* PS and PP, and *U. ohnoi* PP. Blautia species are considered a new wave of potential probiotics as they produce bacteriocins that inhibit colonisation of pathogenic bacteria in the gut. In particular, *Blautia obeum* and *Blautia producta* can inhibit the proliferation of *C. perfringens* and vancomycin-resistant Enterococci [137]. Blautia produce butyric and acetic acid, and are one of the few genera capable of metabolising polyphenols by demethylation of their hydroxyl group [138]. They have also successfully decreased obesity in human trials by regulating G-protein coupled receptors 41 and 43 in gut epithelial cells [139]. 

It was expected that the abundance of Akkermansia (phylum Verrucomicrobiota) would increase in the presence of polyphenols since this genus is capable of metabolising them [140]. Compared to INU (1.15%), Akkermansia abundance did increase when fermented with *P. comosa* PP (2.62%), *E. radiata* PP (2.82%), and *U. ohnoi* PP (3.03%); however, none of the increases was found to be significant; nor were any greater than EGCG (4.00%). Only one species has been identified in the genus (*Akkermansia muciniphila*), and although it forms just 1–4% of the human gut microbiota, its presence is crucial for gut epithelial integrity [141]. *A. muciniphila* is unusual in that it feeds on mucin glycoproteins in the gut epithelial mucus layer. However, it does not degrade the mucus layer but actually strengthens it. As a by-product of mucin digestion *A. muciniphila* produces acetic acid, which supplies energy to epithelial goblet cells that then produce more mucin [142]. All PS and WH seaweed substrates enhanced the abundance of an unclassified phylum termed ‘Bacteria’ compared to INU, while the abundance of another group, the ‘Human Gut Metagenome’, was enhanced by all seaweed ferments (WH, PS, and PP) versus INU. This shows that species richness (total number of species) was enhanced during fermentation with seaweed extracts. In addition, the Shannon Diversity index of all seaweed ferments was significantly greater than INU and EGCG after 24 h, further suggesting that all seaweeds and extracts promoted bacterial numbers and have prebiotic potential.

In the comparison of Firmicutes to Bacteroidetes (Table 9), F/B ratios ranged from 2.301 in *P. comosa* PP to 10.446 in *U. ohnoi* PS, compared to much lower ratios in INU (0.473) and EGCG (1.539). The F/B ratio has been considered a bio-indicator of gut health, although there is no clear consensus on whether this can be applied to all host and microbiota types since the ratio is influenced by factors such as age and body mass [143,144]. Some studies have associated a lower Firmicutes to Bacteroidetes (F/B) ratio with dysbiosis of the gut and impaired immune homeostasis [144,145]. However, in this study, the cellulose negative control and the blank had ratios in the same range as the seaweed extracts (3.228 and 3.484, respectively), so the changes in F/B ratios may not have been due to the effect of seaweed components and could have been influenced by the basal medium. Only two seaweed extracts had greater F/B ratios than cellulose and the blank. These were *U. ohnoi* PS (10.446) and PP (4.712).

The extent to which prebiotics can enhance gut bacterial populations and SCFA production in vitro or in vivo is influenced by several host factors including regular diet, genetics, age, and overall health [146,147]. The bioavailability and bioaccessibility of polysaccharides in human participants after ingestion has been found to vary considerably. Genetic factors that determine the presence or absence of particular digestive enzymes have been investigated in a number of studies [148]. For example, polysaccharide utilisation loci for enzymes that degrade alginate [149,150], laminarin [151], ulvan [118], agar [152], and porphyran [153] have been identified in the genes of marine bacteria. It is hypothesised that these utilisation loci were acquired by human gut bacteria via horizontal transfer over thousands of years of seaweed consumption [149,153]. Pudlo et al. [154] surveyed a global culture collection of 354 human and animal gut bacteria and identified marine bacteria-derived genes involved in seaweed polysaccharide catabolism in 22 species of human gut Bacteroides. These genes were present to a significantly greater extent in Japanese and Chinese subjects, where seaweed forms a regular part of the diet, compared to Northern American and European subjects. A similar geographic distribution was reported by Pluvinage et al. [155] for the presence of genes relating to agarose and porphyran utilisation in human-associated Bacteroides. The study also found that genes involved in laminarin-degrading enzymes (capable of hydrolysing the bonds between *β*(1,3)-linked glucose units) were most abundant [154]. This prevalence of genes related to the metabolism of laminarin, more so than porphyran, alginate, and carrageenan, may be due to the fact that structurally similar *β*-linked glucans occur extensively in many plants and fungi consumed by humans. These findings were further elucidated in a study by Déjean et al. [148] where the human gut microbe *Bacteroides uniformis* was shown to use a single polysaccharide utilisation locus to access *β*(1,3)-linked glucans from laminarin and yeast, and mixed-linkage *β*(1,3)/*β*(1,4)-glucans from cereals. Therefore, people whose gut bacteria are capable of digesting laminarin may also be better equipped to digest other prebiotics such as cereal fibres. This principle has been used to inform in vivo engraftment trials in animals to engineer orthogonal metabolic niches into the gut microbiome that has positively impacted the bioavailability of seaweed polysaccharides. For example, Kearney et al. [156] demonstrated that reversible engraftment of porphyran-utilising *Bacteroides plebeius* is possible with the addition of 1% porphyran to the diet of mice. Similar trials by Shepherd et al. [157] and Pudlo et al. [154] found that mammalian gut bacteria could be transferred by gavage to mice and successfully colonise their gut.

Two potentially negative outcomes of the present study were the increases in abundance of the phyla Proteobacteria and decreases in Actinobacteria, also known as Actinomycetes. All nine seaweed substrates induced significant increases in Proteobacteria compared to INU and EGCG. Increased abundance of Proteobacteria has been found to occur in the gut microbiota of individuals with metabolic disorders and inflammatory bowel disease [158,159,160]. This increase may, in part, be due to the presence of Proteobacteria on the seaweed surface. In their natural marine environment, seaweed surfaces provide an ideal substratum for bacteria and other microorganisms [161]. Molecular identification of marine algal surface bacteria has found Proteobacteria to be one of the most abundant bacterial taxa [162]. If some of these bacteria survived the gastric digestion process, it may account in part for the overall increase in Proteobacteria.

All WH and PS seaweed substrates reduced the abundance of Actinobacteria compared to INU. Decreases in the abundance of Actinobacteria in the gut have been linked to gastrointestinal and systemic diseases [163,164,165]. There were, however, slight increases in Actinobacteria abundance with the *P. comosa*, *E. radiata*, and *U. ohnoi* polyphenol extracts compared to EGCG, but they were not significant. There was one exception within the phylum Actinobacteria. The order Bifidobacteria of the phylum Actinobacteria was significantly enhanced by *P. comosa* PP (42.01%) compared to EGCG (33.30%) (*p* = 0.0109). This may be due to *P. comosa* PP’s having the highest concentration of phlorotannins (4.33 ± 0.15 mg PE/g) amongst the three seaweeds, which have been reported to exert a prebiotic effect on the abundance of Bifidobacteria and Lactobacillus [166] in the same way that plant-derived polyphenols do [167]. The inability of the polyphenol extracts to significantly enhance bacterial abundance in any of the Firmicutes, Bacteroidetes, or Verrucomicrobiota suggests that algal polyphenols may not be suitable substrates for the majority of the bacterial population, but were successful in promoting several niche groups such as Bifidobacteria, Blautia (*E. radiata* PP only vs. *B. hydrogenotrophica* (p = 0.04372), and Barnesiella.

Another factor that may be considered anti-nutritional was the reduction of abundance within the phylum Euryarchaeota, domain Archaea, by eight of the nine seaweed substrates compared to the cellulose negative control and the blank (Supplementary Figure S25). *U. ohnoi* PS, which induced an increase (*p* = 0.01646) compared to the blank, was the exception. Methanobrevibacter was the only genus sequenced in this phylum (no species specified). It is the predominant archaeon of the human gut, with *M. smithii* being the most common species [168]. Methanobrevibacter have important symbiotic roles in the human gut including the conversion of bacterial-produced H_2_ into methane [169], allowing for a more complete oxidation of food substrates, which increases energy harvest and the production of SCFA and adenosine triphosphate [170]. The reduction of Methanobrevibacter abundance was most likely due to the bromine content of the seaweeds, which competitively inhibits the activity of the archaeal enzyme methyl-coenzyme M reductase [171,172]. Bromine ranged from 0.35 mg/g in *P. comosa*, 0.34 mg/g in *E. radiata*, to 0.06 mg/g *U. ohnoi*. Since *U. ohnoi* contained the least bromine, this may be the reason it did not inhibit Methanobrevibacter growth. The inhibition of methanogens was not an aim of this study; however, the findings may be useful for further investigations where methane reduction is the intent. 

Furthermore, there are a number of limitations that affect any conclusions drawn from this study. Effects determined in vitro are only putative until proven in vivo. Simulated digestion models are not fully representative of the living gut or the biological fate of dietary components since in vitro models lack in vivo factors such as first pass effect, renal clearance, and metabolisation by intestinal epithelial cells [32]. For example, in an in vivo study, the polyphenol extracts would undergo biotransformation in the liver and re-enter the gastrointestinal tract in bile via enterohepatic recirculation as conjugated polyphenol compounds with different biological effects [173]. The small sample size reduces the statistical power of this study, which may affect the margin of error. Moreover, the pooled faecal inoculum from three individuals may not be representative of the gut microbiota of the broader population. 

## 5. Conclusions

Seaweeds are an underutilised, relatively inexpensive, and sustainable source of prebiotics. This study shows that fibre- and polyphenol-rich substrates can significantly enhance the abundance of many commensal bacteria and their production of SCFAs after 24 h in vitro. Whole *P. comosa*, *E. radiata*, *U. ohnoi*, and their polysaccharide extracts induced up to three-fold increases in total and individual SCFAs, and more than doubled the abundance of beneficial, butyrate-producing bacterial taxa. In addition, bacterial species richness and diversity was significantly increased. Prebiotic food extracts from raw seaweed biomass could potentially benefit harvesters and seaweed processors through the development of value-added products. Although clinical trials are required to confirm any in vitro effects, consuming whole seaweeds and their polysaccharide and polyphenol extracts may have potential for use as food supplements to support gut health and impact inflammation.

## Figures and Tables

**Table 1:** Proximate composition of WH seaweeds and PS and PP extracts (percentage ± SD).

	(% of DW)					
	*P. comosa* WH	*E. radiata* WH	*U. ohnoi* WH	*P. comosa* PS	*E. radiata* PS	*U. ohnoi* PS
Moisture	6.04 ± 0.77	5.59 ± 0.87	4.73 ± 1.02			
Ash	18.06 ± 1.47	24.73 ± 1.58	15.61 ± 1.92			
Protein	3.67 ± 0.56	6.02 ± 0.06	19.28 ± 0.05			
Lipids	1.96 ± 0.14	2.91 ± 0.08	3.04 ± 0.32			
Insoluble fibre	37.41 ± 0.72	27.82 ± 0.21	32.01 ± 0.34	50.49 ± 1.81	63.09 ± 1.74	59.61 ± 1.17
Soluble fibre	23.47 ± 0.60	13.46 ± 0.45	15.05 ± 0.48	31.37 ± 0.56	30.86 ± 0.96	28.32 ± 0.30
Total polysaccharides *	62.53 ± 1.38	58.85 ± 1.49	50.14 ± 0.87	83.71 ± 2.32	94.56 ± 2.07	91.35 ± 1.86

* Total polysaccharides were quantified by the Dubois phenol sulphuric acid method. Soluble and insoluble fibre was quantified by the enzymatic gravimetric method.

**Table 2:** Total phlorotannin content of *P. comosa* and *E. radiata* and total polyphenol content of *U. ohnoi*.

	*P. comosa*	*E. radiata*	*U. ohnoi*
	(mg PE/g)		(mg GAE/g)
WH seaweeds	0.38 ± 0.02	0.55 ± 0.03	0.35 ± 0.02
PP extracts	4.33 ± 0.15	3.48 ± 0.27	2.46 ± 0.21

**Table 3:** Total neutral non-starch polysaccharides content of WH seaweeds and their PS extracts (DW) (percentage ± SD).

	Rhamnose	Fucose	Ribose	Arabinose	Xylose	Mannose	Galactose	Glucose	
	Soluble NNSP (% DW)								Total Soluble NNSP (% DW)
*P. comosa* WH	0.00 ± 0.00	3.31 ± 0.14	0.00 ± 0.00	0.00 ± 0.00	1.34 ± 0.07	0.59 ± 0.05	0.52 ± 0.03	0.25 ± 0.00	6.00
*P. comosa* PS	0.00 ± 0.00	5.05 ± 0.21	0.00 ± 0.00	0.00 ± 0.00	2.59 ± 0.13	1.09 ± 0.07	0.92 ± 0.08	0.52 ± 0.01	10.17
*E. radiata* WH	0.00 ± 0.00	0.98 ± 0.03	0.00 ± 0.00	0.00 ± 0.00	0.21 ± 0.01	0.04 ± 0.00	0.31 ± 0.00	0.39 ± 0.00	1.93
*E. radiata* PS	0.00 ± 0.00	1.39 ± 0.08	0.00 ± 0.00	0.00 ± 0.00	0.35 ± 0.01	0.08 ± 0.00	0.59 ± 0.02	0.61 ± 0.03	3.02
*U. ohnoi* WH	0.58 ± 0.06	0.00 ± 0.00	0.09 ± 0.01	0.00 ± 0.00	0.10 ± 0.00	0.09 ± 0.01	0.02 ± 0.00	0.00 ± 0.00	0.89
*U. ohnoi* PS	1.12 ± 0.09	0.00 ± 0.00	0.16 ± 0.02	0.00 ± 0.00	0.17 ± 0.01	0.17 ± 0.02	0.04 ± 0.00	0.00 ± 0.00	1.66
	**Insoluble NNSP (% DW)**								**Total insoluble NNSP (% DW)**
*P. comosa* WH	0.00 ± 0.00	1.91 ± 0.05	0.00 ± 0.00	0.00 ± 0.00	0.65 ± 0.03	0.16 ± 0.00	0.00 ± 0.00	3.36 ± 0.27	6.08
*P. comosa* PS	0.00 ± 0.00	3.32 ± 0.07	0.00 ± 0.00	0.00 ± 0.00	1.17 ± 0.14	0.28 ± 0.01	0.00 ± 0.00	6.14 ± 0.58	10.91
*E. radiata* WH	0.00 ± 0.00	0.00 ± 0.00	0.00 ± 0.00	0.00 ± 0.00	0.00 ± 0.00	0.00 ± 0.00	0.00 ± 0.00	2.60 ± 0.09	2.60
*E. radiata* PS	0.00 ± 0.00	0.00 ± 0.00	0.00 ± 0.00	0.00 ± 0.00	0.00 ± 0.00	0.00 ± 0.00	0.00 ± 0.00	4.72 ± 0.13	4.72
*U. ohnoi* WH	3.04 ± 0.09	0.00 ± 0.00	0.00 ± 0.00	0.00 ± 0.00	1.20 ± 0.06	0.00 ± 0.00	0.00 ± 0.00	2.75 ± 0.06	6.99
*U. ohnoi* PS	4.01 ± 0.00	0.00 ± 0.00	0.00 ± 0.00	0.00 ± 0.00	2.28 ± 0.09	0.00 ± 0.00	0.00 ± 0.00	3.68 ± 0.17	9.98

**Table 4:** Essential mineral content of whole seaweeds *.

	*P. comosa*	*E. radiata*	*U. ohnoi*
Mineral	mg/g (DW)		
Bromine	0.35	0.34	0.06
Calcium	13.00	11.20	2.79
Chloride	47.7	101.0	47.5
Chromium	0.00031	0.0032	0.00043
Copper	0.00014	0.00093	0.013
Iodine	1.70	3.40	0.0029
Iron	0.023	0.88	0.19
Magnesium	7.22	5.76	32.00
Manganese	0.0095	0.0074	0.017
Molybdenum	0.00035	0.00031	0.00023
Phosphorus	1.46	1.45	2.17
Potassium	68.10	78.00	25.00
Selenium	<0.005	<0.005	<0.005
Sodium	26.3	30.2	23.1
Zinc	0.029	0.019	0.041

* Data generated by the National Measurement Institute, Melbourne was provided as the mean of three values, without standard deviation.

**Table 5:** Oxygen radical absorbance capacity of WH seaweeds and PP extracts (ORAC units µmol TE/g DW ± SD).

	µmol TE/g (DW)	
	Whole	Polyphenol Extract
*P. comosa*	84.5 ± 2.32	224.7 ± 5.33
*E. radiata*	111.0 ± 2.46	345.4 ± 6.87
*U. ohnoi*	17.5 ± 1.05	59.1 ± 1.96

**Table 6:** Percentage of WH seaweed and PS extracts digested after simulated gastric digestion (percentage ± SD).

Seaweed	Gastrically Digested Portion (% DW)
*P. comosa* WH	20.28 ± 0.93
*E. radiata* WH	41.98 ± 1.84
*U. ohnoi* WH	26.90 ± 1.07
*P. comosa* PS	9.36 ± 0.88
*E. radiata* PS	11.77 ± 1.94
*U. ohnoi* PS	8.38 ± 1.53

**Table 7:** Total and individual short chain fatty acid concentration (µmol/mL ± SD) after 24 h fermentation *.

Substrate	Total SCFA	Acetic	Butyric	Propionic	iso-Butyric	iso-Valeric	Valeric	Caproic
Blank	33.53 ± 2.03 ^b^	18.33 ± 1.29 ^b^	5.17 ± 0.37 ^a^	5.29 ± 0.19 ^b^	1.17 ± 0.11 ^a^	1.80 ± 0.05 ^b^	1.76 ± 0.02 ^a^	0.00 ± 0.00 ^a^
Cellulose	35.55 ± 1.45 ^b^	18.37 ± 1.02 ^b^	5.87 ± 0.19 ^a^	5.91 ± 0.10 ^b^	1.42 ± 0.02 ^a^	2.06 ± 0.05 ^b^	1.92 ± 0.05 ^a^	0.00 ± 0.00 ^a^
Inulin	71.05 ± 1.08 ^d^	50.33 ± 0.77 ^c^	7.32 ± 0.06 ^a^	13.00 ± 0.25 ^d^	0.00 ± 0.00 ^a^	0.00 ± 0.00 ^a^	0.40 ± 0.01 ^a^	0.00 ± 0.00 ^a^
EGCG	7.76 ± 0.09 ^a^	5.72 ± 0.06 ^a^	0.99 ± 0.02 ^a^	1.06 ± 0.01 ^a^	0.00 ± 0.00 ^a^	0.00 ± 0.00 ^a^	0.00 ± 0.00 ^a^	0.00 ± 0.00 ^a^
*P. comosa* WH	52.32 ± 0.87 ^bc^	32.68 ± 0.46 ^bc^	8.11 ± 0.28 ^a^	6.78 ± 0.08 ^b^	0.94 ± 0.03 ^a^	1.49 ± 0.02 ^b^	2.09 ± 0.00 ^a^	0.23 ± 0.00 ^a^
*P. comosa* PS	37.38 ± 0.74 ^b^	24.27 ± 0.23 ^b^	4.64 ± 0.12 ^a^	6.53 ± 0.07 ^b^	0.30 ± 0.26 ^a^	0.71 ± 0.03 ^ab^	0.71 ± 0.03 ^a^	0.22 ± 0.01 ^a^
*P. comosa* PP	49.50 ± 1.28 ^bc^	24.30 ± 0.48 ^b^	13.71 ± 0.58 ^ab^	9.97 ± 0.15 ^c^	0.00 ± 0.00 ^a^	0.45 ± 0.01 ^ab^	1.08 ± 0.05 ^a^	0.00 ± 0.00 ^a^
*E. radiata* WH	61.20 ±1.02 ^cd^	37.56 ± 0.75 ^bc^	9.45 ± 0.43 ^ab^	8.96 ± 0.34 ^c^	1.00 ± 0.09 ^a^	1.65 ± 0.14 ^b^	2.27 ± 0.01 ^a^	0.29 ± 0.01 ^a^
*E. radiata* PS	227.53 ± 5.39 ^g^	145.03 ± 4.38 ^g^	30.04 ± 0.40 ^c^	31.35 ± 0.25 ^f^	4.40 ± 0.10 ^b^	6.60 ± 0.13 ^c^	8.58 ± 0.07 ^c^	1.53 ± 0.06 ^c^
*E. radiata* PP	183.73 ± 20.06 ^f^	99.44 ± 1.62 ^e^	44.87 ± 11.22 ^d^	28.62 ± 2.10 ^f^	0.97 ± 1.68 ^a^	2.74 ± 1.59 ^b^	6.09 ± 1.79 ^bc^	0.99 ± 0.07 ^b^
*U. ohnoi* WH	208.68 ± 19.08 ^fg^	119.52 ± 12.28 ^f^	33.07 ± 1.87 ^c^	32.69 ± 1.99 ^f^	4.95 ± 0.77 ^b^	8.07 ± 0.89 ^d^	10.12 ± 0.83 ^c^	0.26 ± 0.45 ^a^
*U. ohnoi* PS	182.91 ± 3.47 ^f^	104.83 ± 2.39 ^e^	27.88 ± 0.37 ^c^	28.78 ± 0.25 ^f^	4.38 ± 0.14 ^b^	6.96 ± 0.16 ^c^	8.98 ± 0.11 ^c^	1.10 ± 0.06 ^b^
*U. ohnoi* PP	140.42 ± 2.93 ^e^	77.07 ± 1.58 ^d^	27.13 ± 0.13 ^c^	29.47 ± 0.89 ^f^	0.00 ± 0.00 ^a^	2.05 ± 0.09 ^b^	4.70 ± 0.23 ^b^	0.00 ± 0.00 ^a^

* Letters indicate a significant difference (*p* ≤ 0.05) between means within the same column.

**Table 9:** Ratio of Firmicutes/Bacteroidetes in seaweed ferments, INU, EGCG or cellulose standards, and the blank at 24 h.

	**WH**	**PS**	**PP**
*P. comosa*	0.794/0.332 (2.391)	0.757/0.247 (3.065)	0.573/0.249 (2.301)
*E. radiata*	0.816/0.334 (2.443)	0.804/0.319 (2.520)	0.655/0.245 (2.673)
*U. ohnoi*	0.777/0.252 (3.083)	0.773/0.074 (10.446)	0.688/0.146 (4.712)
**Controls**			
Inulin	0.325/0.687 (0.473)		
EGCG	0.671/0.436 (1.539)		
Cellulose	0.778/0.241(3.228)		
Blank	0.763/0.219 (3.484)		

## Supplementary Materials

The following supporting information can be downloaded at: https://www.mdpi.com/article/10.3390/nu14102163/s1, Figure S1. Total short chain fatty acid production by bacteria after 24 h fermentation with *U. ohnoi*, *E. radiata* or *P. comosa* whole, polysaccharide or polyphenol extracts, compared to SCFA production by bacteria fermented with inulin, epigallocatechin gallate, cellulose, or basal media only (blank). Figure S2. Heat map of *P. comosa* influence on bacterial family abundance versus INU and EGCG controls. Figure S3. Heat map of E. radiata influence on bacterial family abundance versus INU and EGCG controls. Figure S4. Heat map of *U. ohnoi* influence on bacterial family abundance versus INU and EGCG controls. Figure S5. U. ohnoi WH enhanced phylum Firmicutes (P = 0) vs INU. Figure S6. *E. radiata* WH enhanced phylum Firmicutes (P = 0) vs. INU. Figure S7. *P. comosa* PS enhanced phylum Firmicutes (P = 0) vs. INU. Figure S8. *U. ohnoi* PP enhanced phylum Firmicutes (P = 0) vs INU. Figure S9. *P. comosa* PP was the only sample that reduced phylum Firmicutes (P = 0.0093) vs EGCG. Figure S10. All WH, PS and PP seaweed substrates enhanced the abundance of order Lactobacillales compared to INU. Figure S11. Genus Streptococcus increased significantly compared to INU in all WH, PS and PP extracts. Figure S12. The abundance of the family Eubacteriaceae was enhanced by all WH, PS, and PP extracts compared to INU and EGCG (with the exception of *E. radiata* PP vs EGCG where there was no significant increase). Figure S13. The abundance of the species Eubacterium halii was enhanced significantly by all WH, PS, and PP extracts compared to INU. Figure S14. Genus Faecalibacterium increased significantly compared to INU in all WH, PS and PP extracts. Figure S15. Compared to INU, genus Butyricicoccus was enhanced by all WH and PS ferments, but decreased compared to EGCG. Figure S16. Genus Roseburia was enhanced by all WH and PS ferments but not by PP. Figure S17. (A) U. ohnoi PS (P = 0.00007), (B) *P. comosa* WH (P = 0.00087) and (C) E. radiata PP (P = 0.0233) enhanced Blautia hydrogenotrophica abundance compared to INU. Figure S18. Genus Akkermansia was enhanced by all WH and PS ferments but not by PP. Figure S19. *P. comosa* PP was the only seaweed substrate to enhance Bifidobacteria compared to the EGCG control (P = 0.00648). Figure S20. All PS and WH seaweed substrates increased the abundance of the phylum of unclassified ‘Bacteria’ vs INU suggesting that species richness was enhanced. Figure S21. Human gut metagenome abundance was enhanced by all nine seaweed ferments vs INU. Figure S22. Shannon diversity index increased in all seaweed ferments vs INU. Figure S23. Intestinimonas butyriciproducens of the phylum Firmicutes was enhanced by *U. ohnoi* WH vs. INU (P = 0). Figure S24. Genus Barnesiella abundance was enhanced in all WH, PS and PP ferments compared to INU and EGCG. Figure S25. The abundance of Methanobrevibacter was reduced by all nine seaweed ferments vs the blank and cellulose.
