# Supplementary material for: The Prebiotic Effect of Australian Seaweeds on Commensal Bacteria and Short Chain Fatty Acid Production in a Simulated Gut Model
Source: Nutrients. 2022 May 23;14(10):2163. doi: 10.3390/nu14102163 (PMC9146517; doi:10.3390/nu14102163)
Supplement: Supplementary file 1 [file nutrients-14-02163-s001.zip › nutrients-1733549-supplementary.pdf]

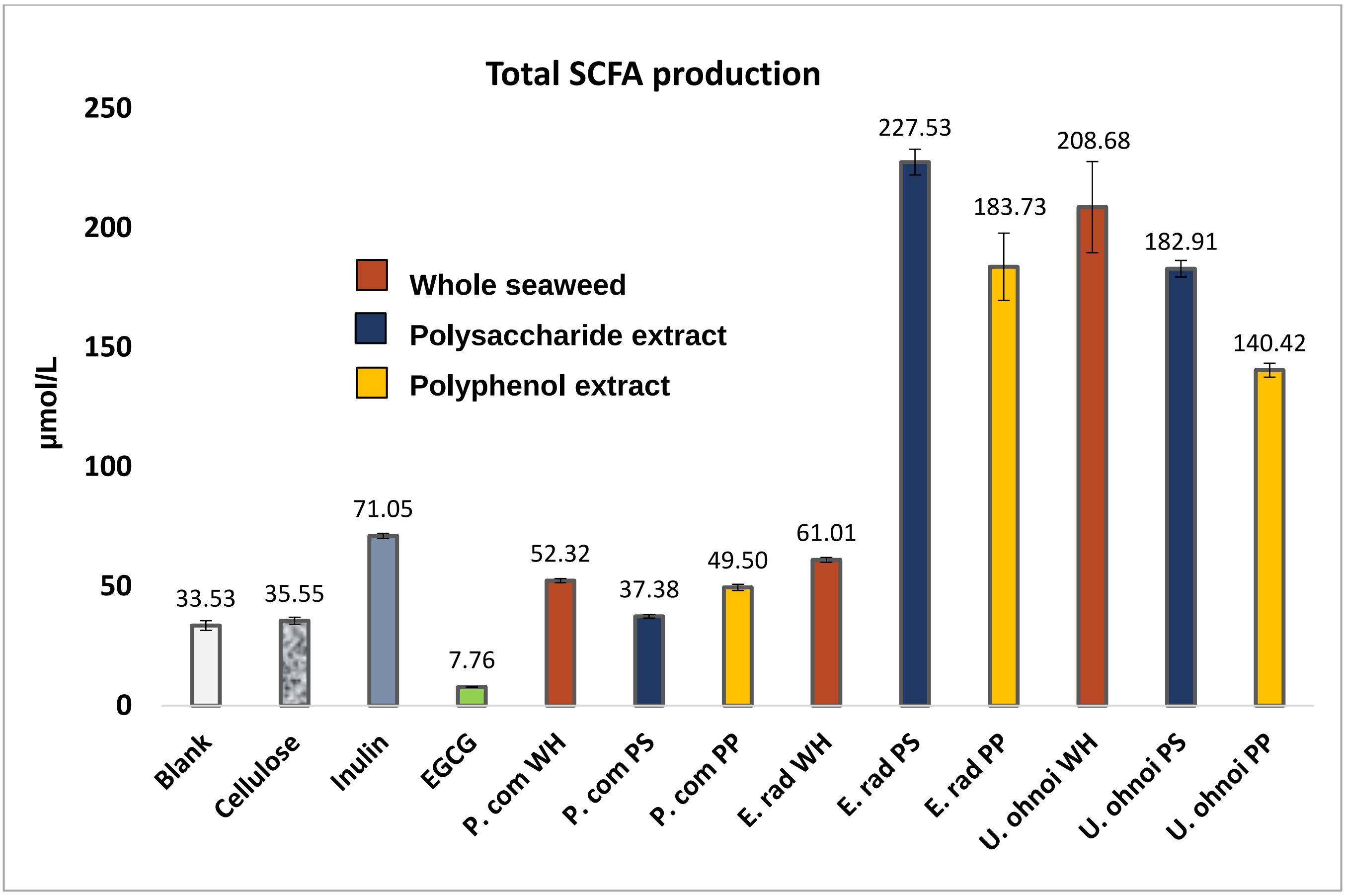


Figure S1. Total short chain fatty acid production by bacteria after 24 hr fermentation with *U. ohnoi*, *E. radiata* or *P. comosa* whole, polysaccharide or polyphenol extracts, compared to SCFA production by bacteria fermented with inulin, epigallocatechin gallate, cellulose, or basal media only (blank).

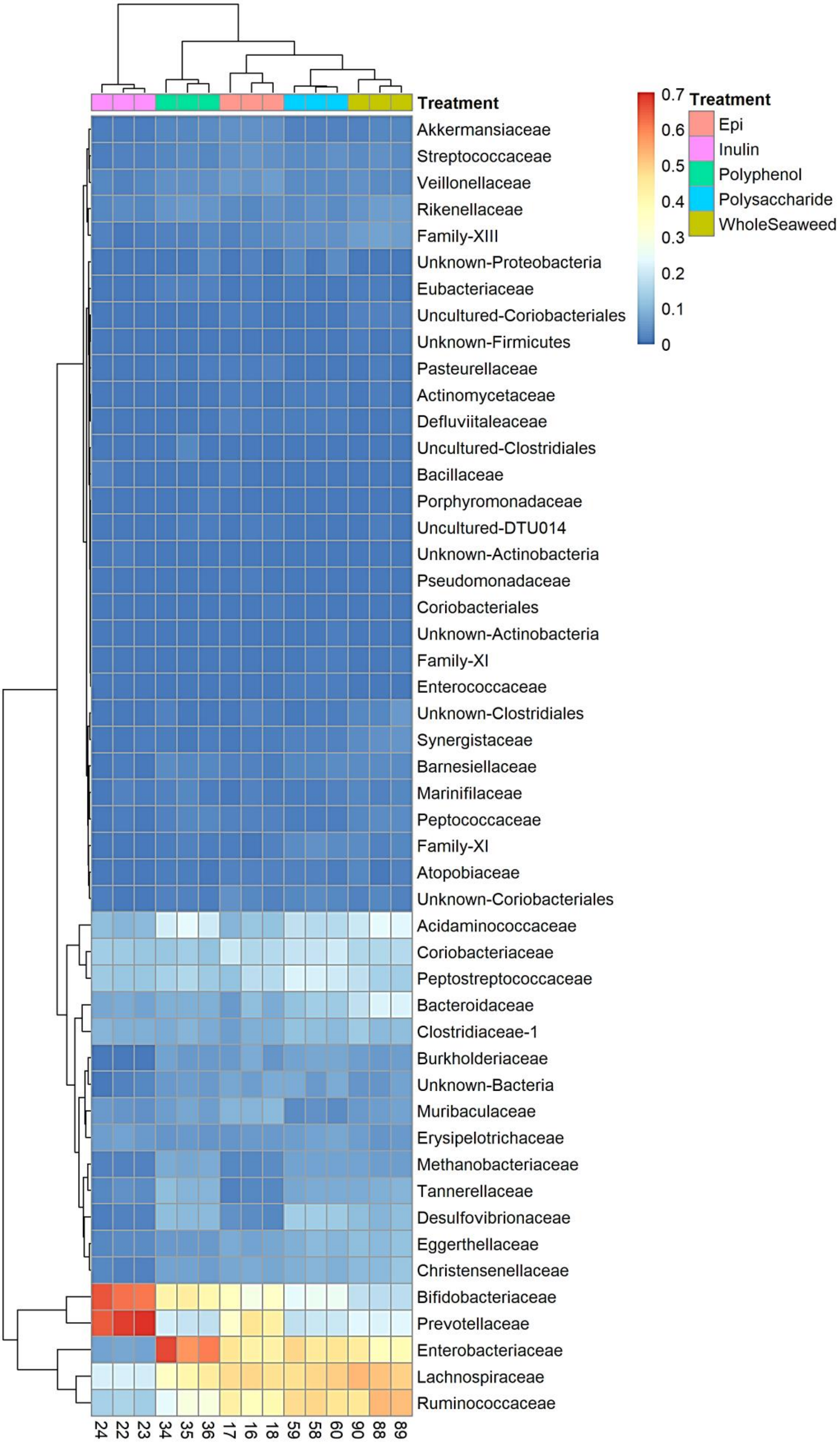


Figure S2. Heat map of *P. comosa* influence on bacterial family abundance versus INU and EGCG controls

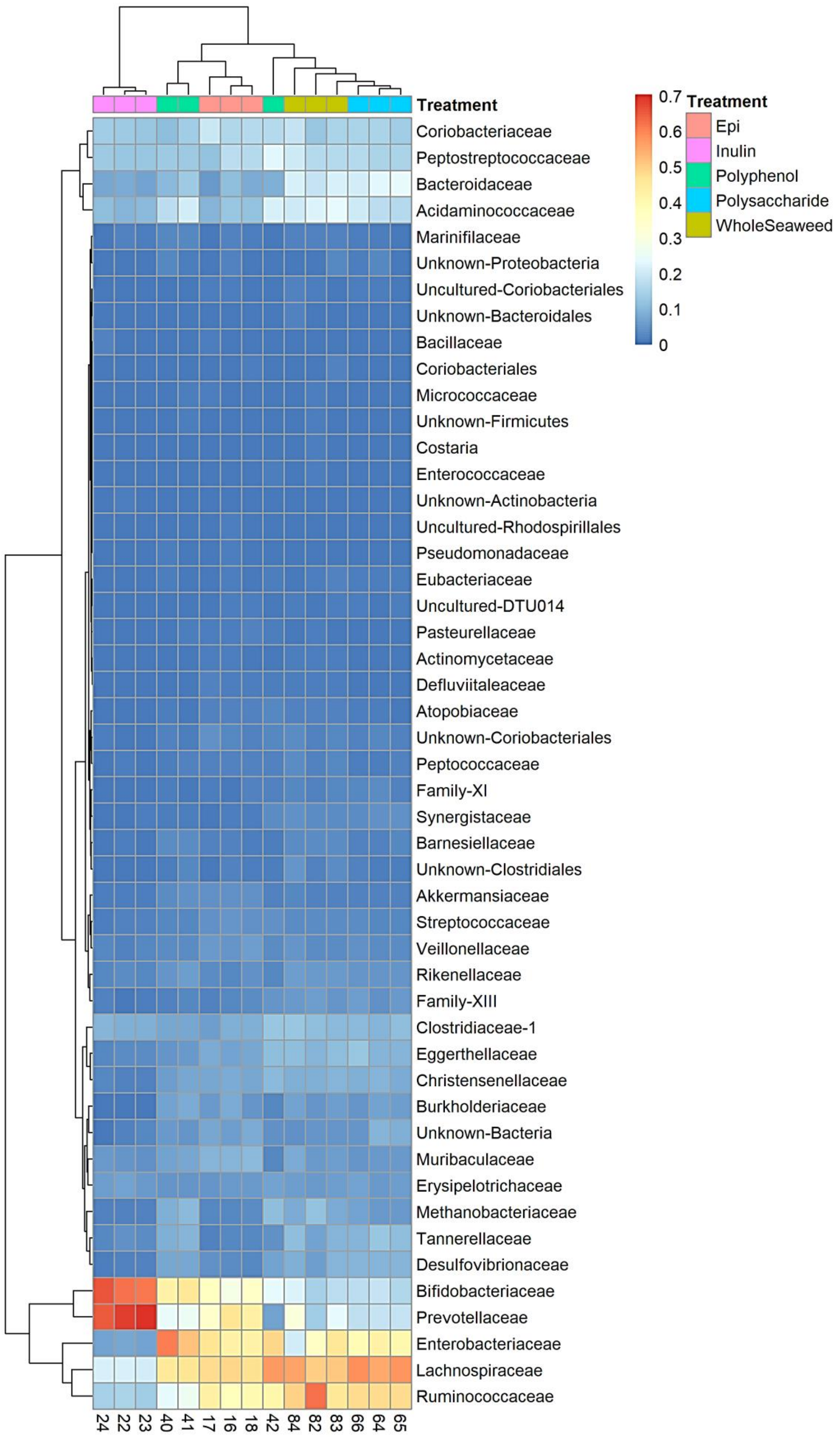


Figure S3. Heat map of *E. radiata* influence on bacterial family abundance versus INU and EGCG controls.

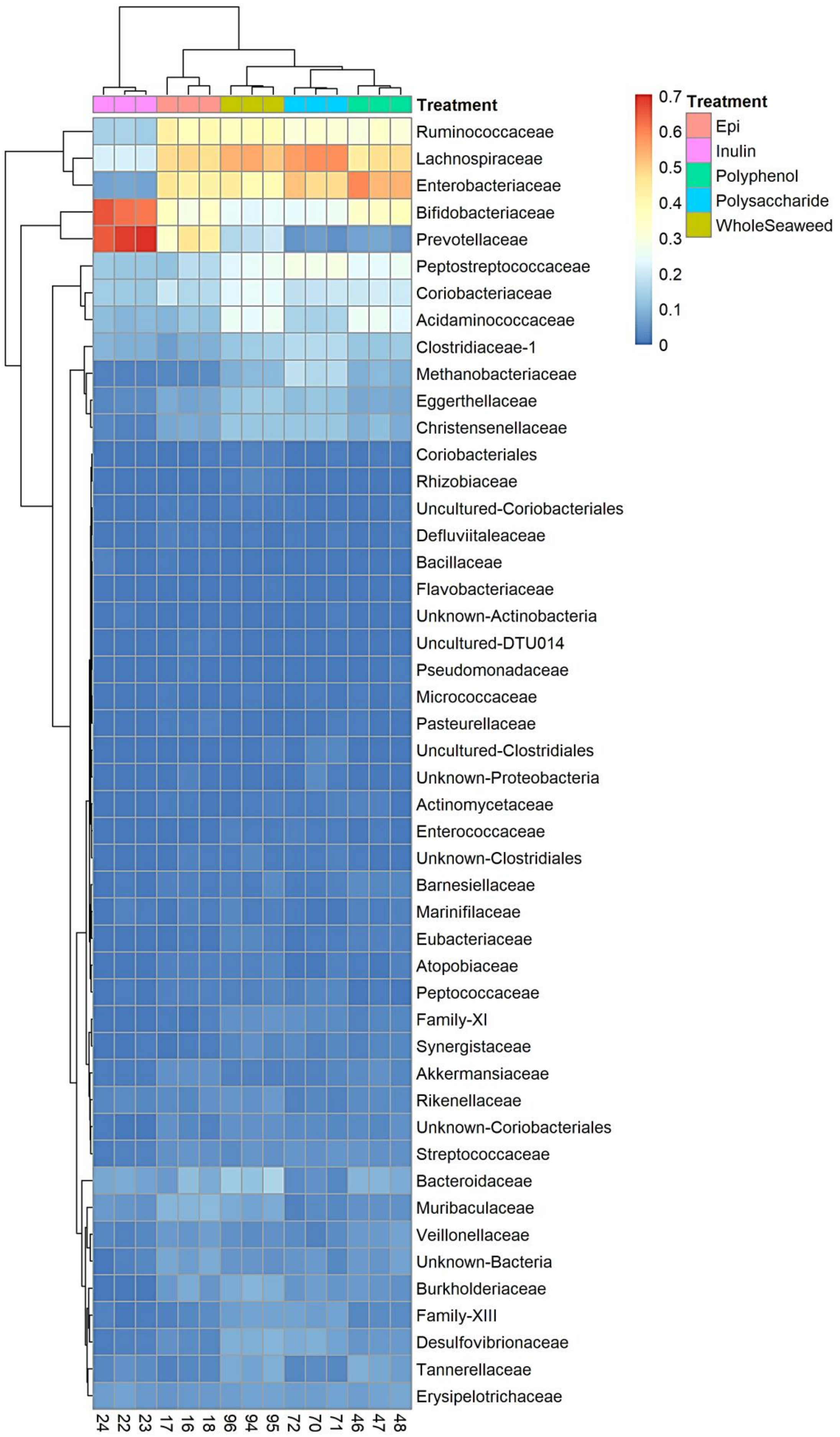


Figure S4. Heat map of *U. ohnoi* influence on bacterial family abundance versus INU and EGCG controls.

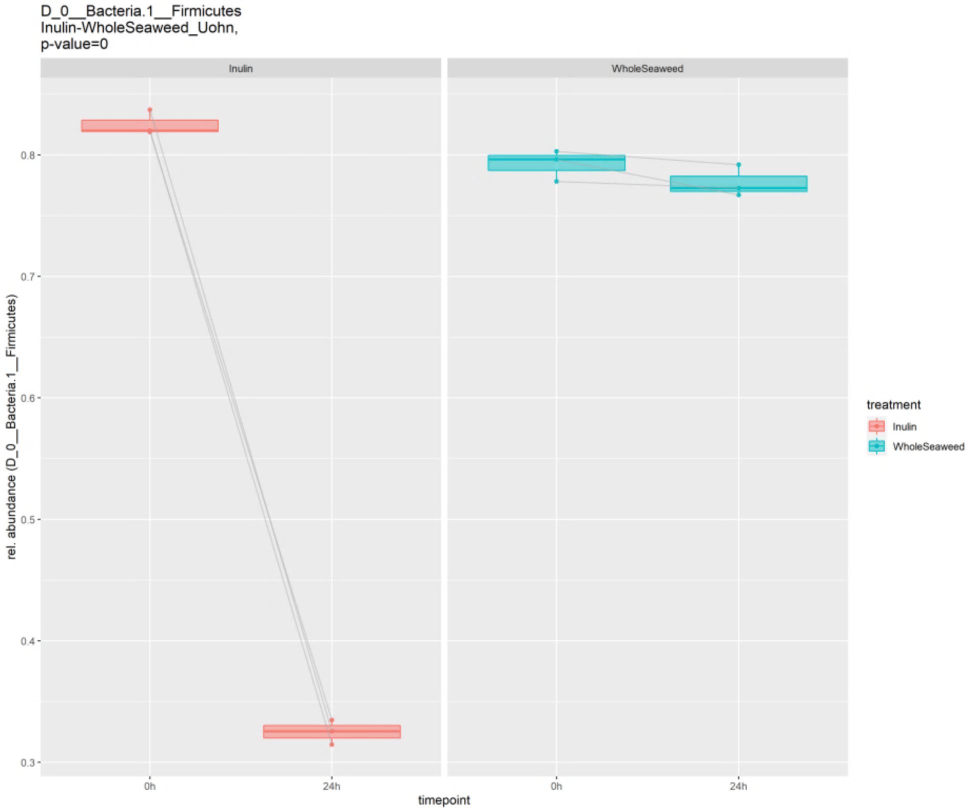


Figure S5. *U. ohnoi* WH enhanced phylum Firmicutes (P = 0) *vs* INU

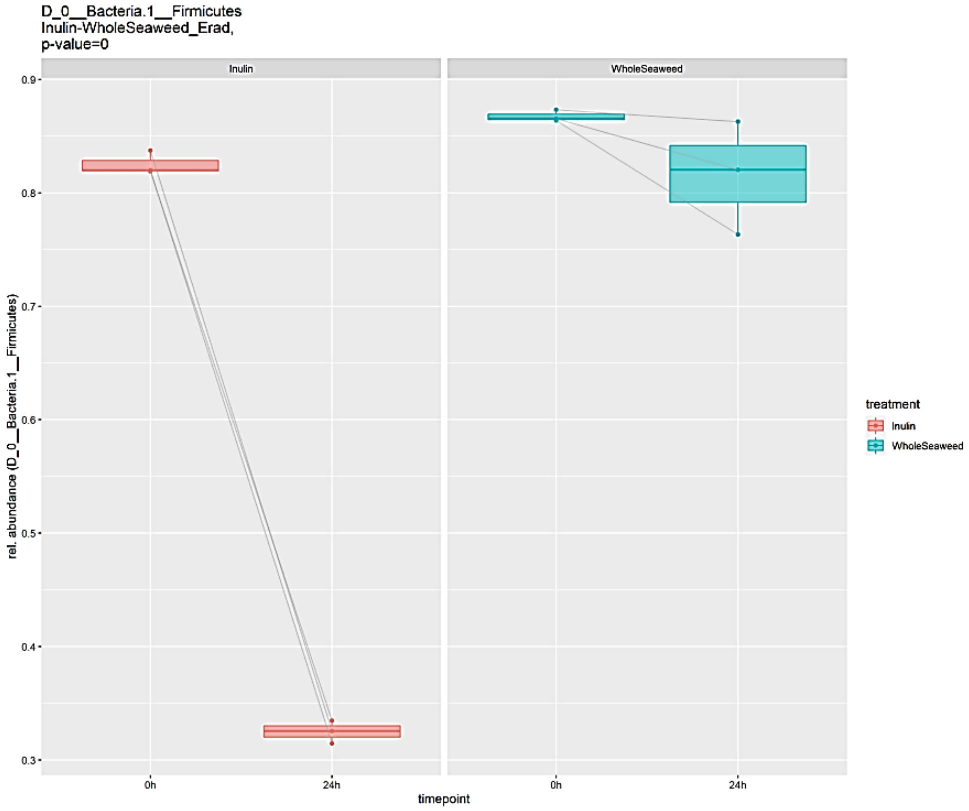


Figure S6. *E. radiata* WH enhanced phylum Firmicutes (P = 0) *vs* INU

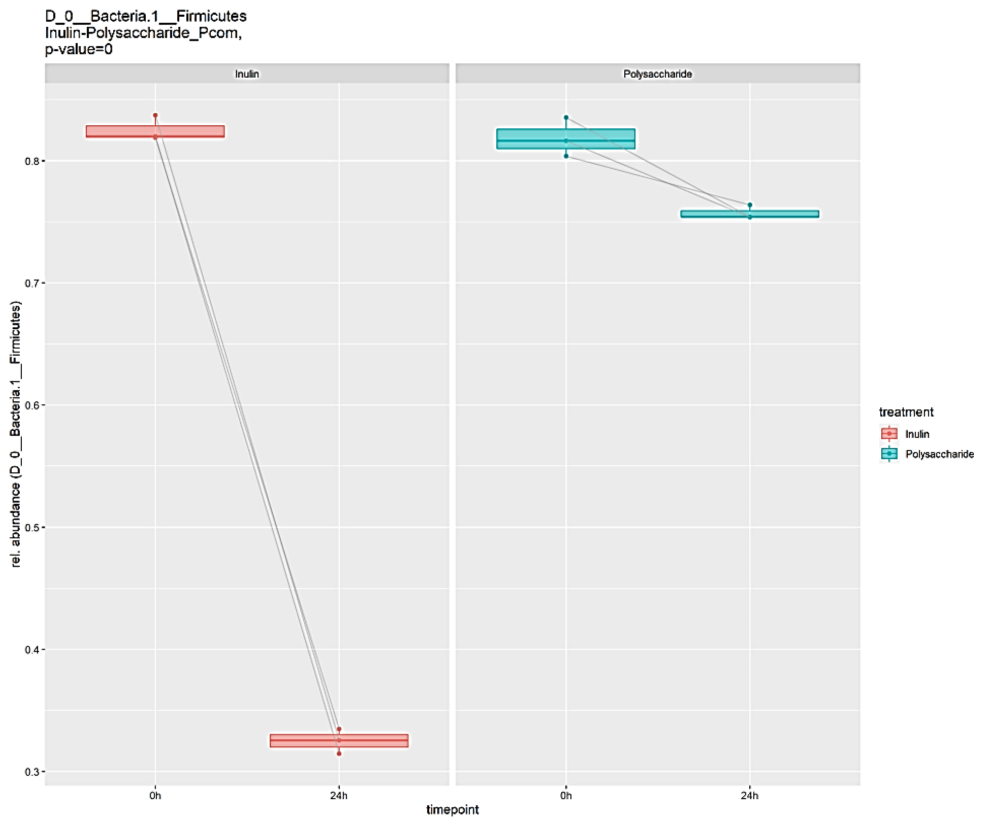


Figure S7. *P. comosa* PS enhanced phylum Firmicutes (P = 0) *vs* INU

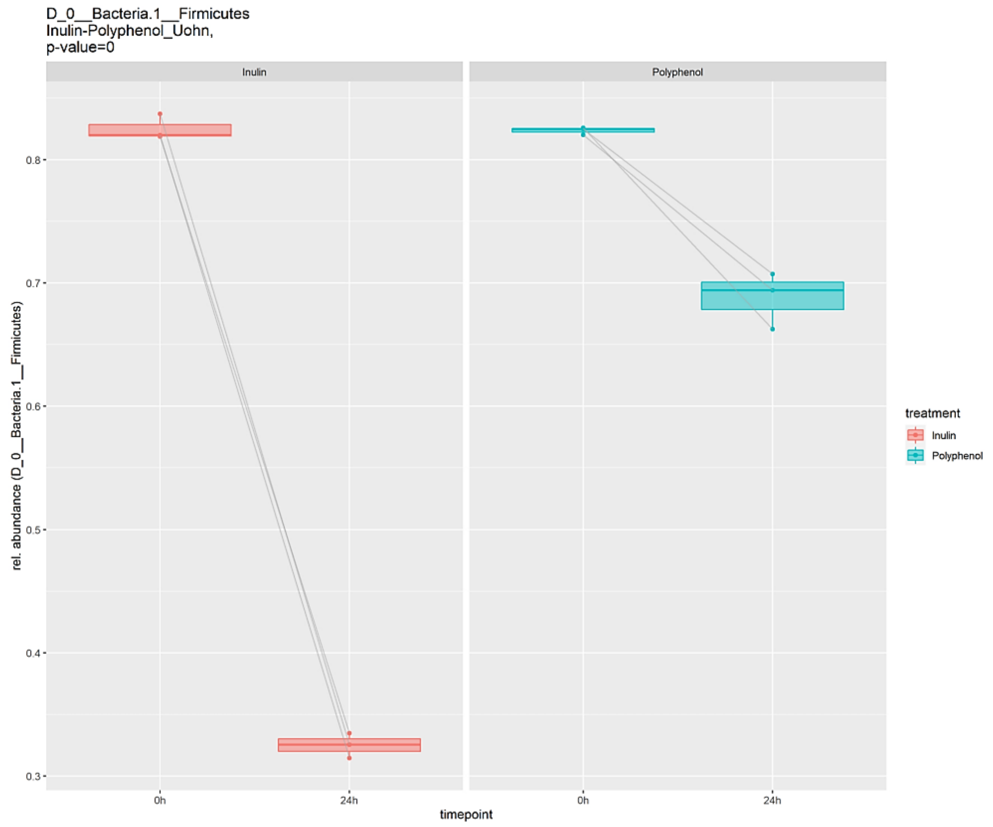


Figure S8. *U. ohnoi* PP enhanced phylum Firmicutes (P = 0) *vs* INU

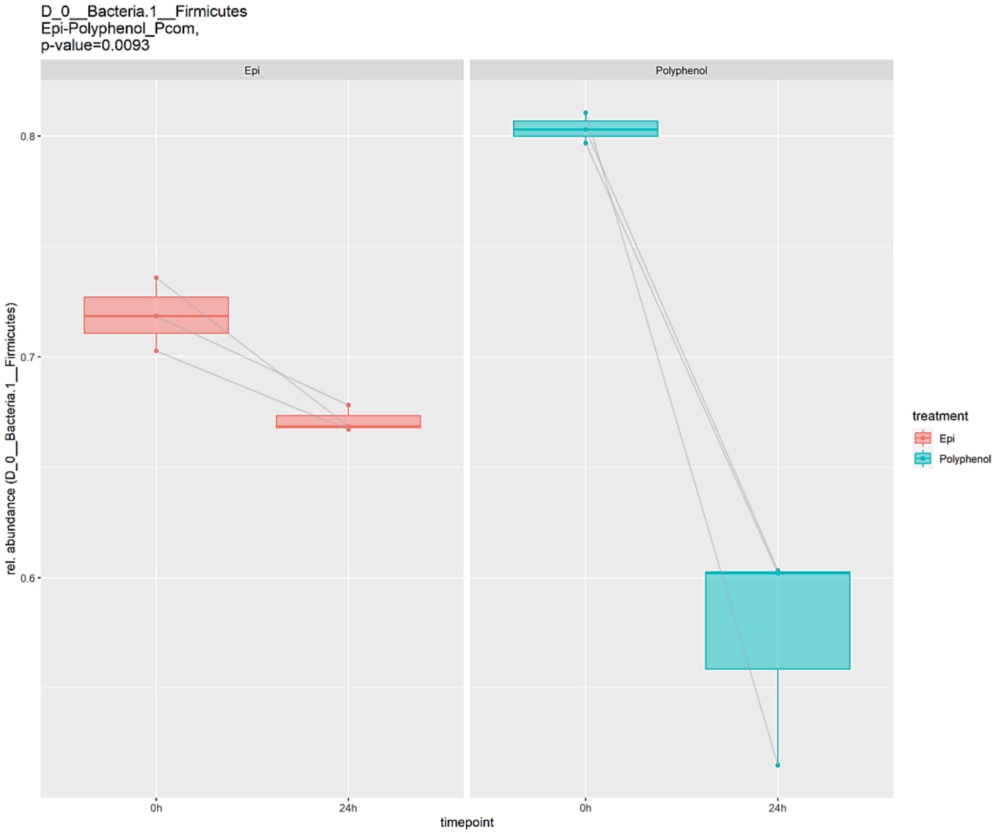


Figure S9. *P. comosa* PP was the only sample that reduced phylum Firmicutes (P = 0.0093) *vs* EGCG

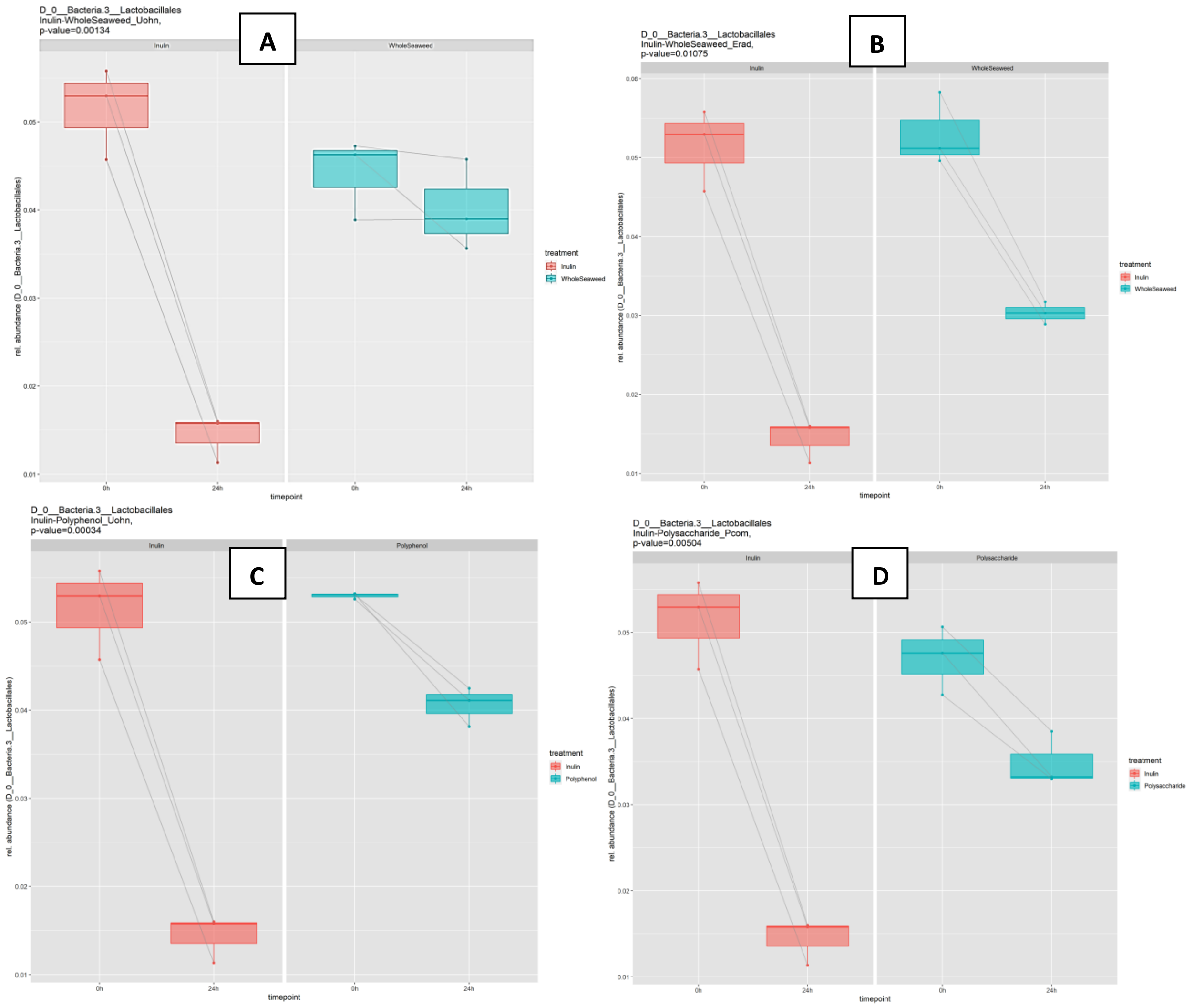


Figure S10. All WH, PS and PP seaweed substrates enhanced the abundance of order Lactobacillales compared to INU. Examples shown are (A) *U. ohnoi* WH (P = 0.00134), (B) *E. radiata* WH (P = 0.01075), (C) *U. ohnoi* PP (P = 0.00034) and (D) *P. comosa* PS (P = 0.00504) *vs* INU.

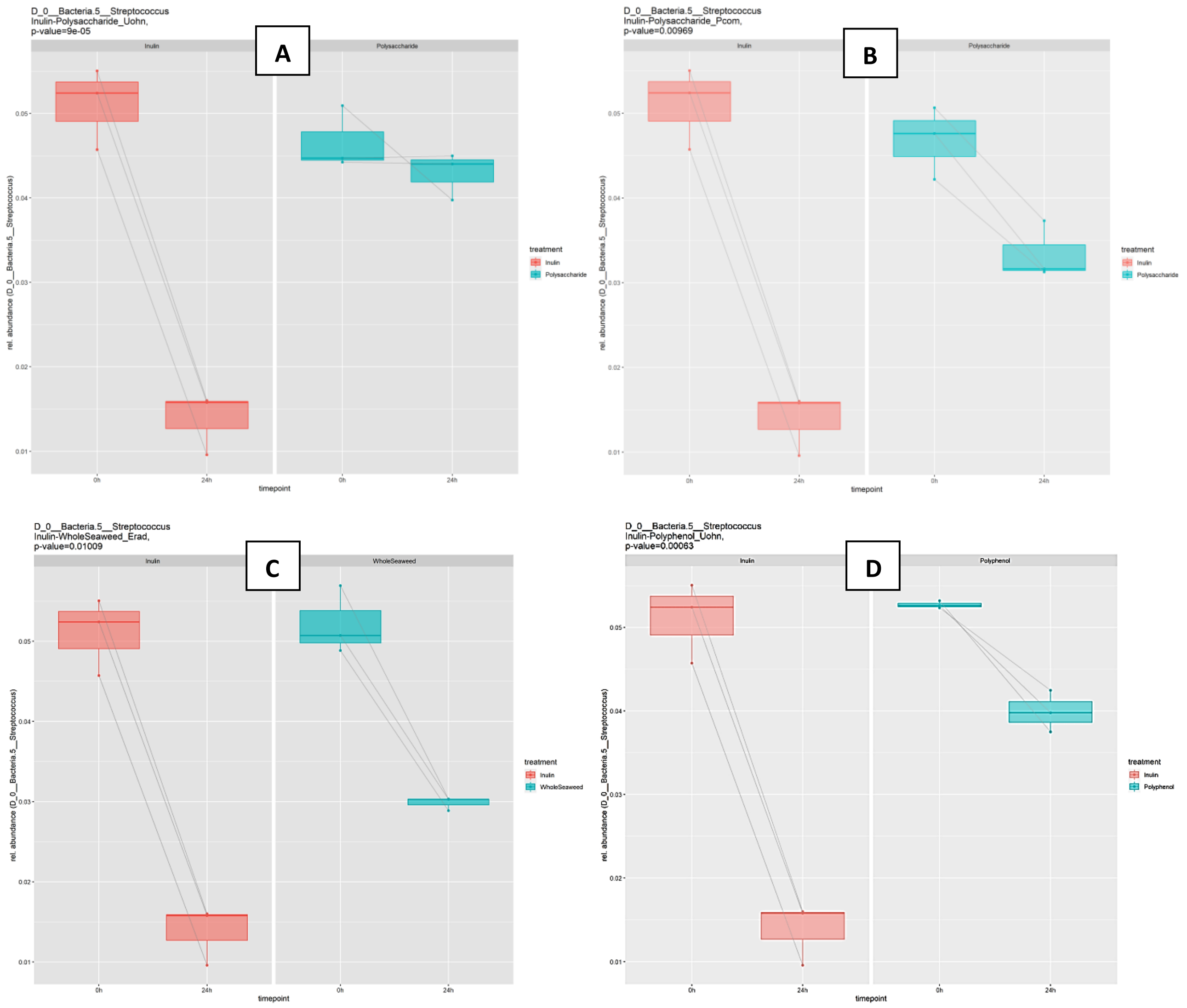


Figure S11. Genus Streptococcus increased significantly compared to INU in all WH, PS and PP extracts. Examples of enhancement shown are (A) *U. ohnoi* PS (P = $0.05x10^{-9}$), (B) *P. comosa* PS (P = 0.00969), (C) *E. radiata* WH (P = 0.01009) and (D) *U. ohnoi* PP (P = 0.00063) *vs* INU

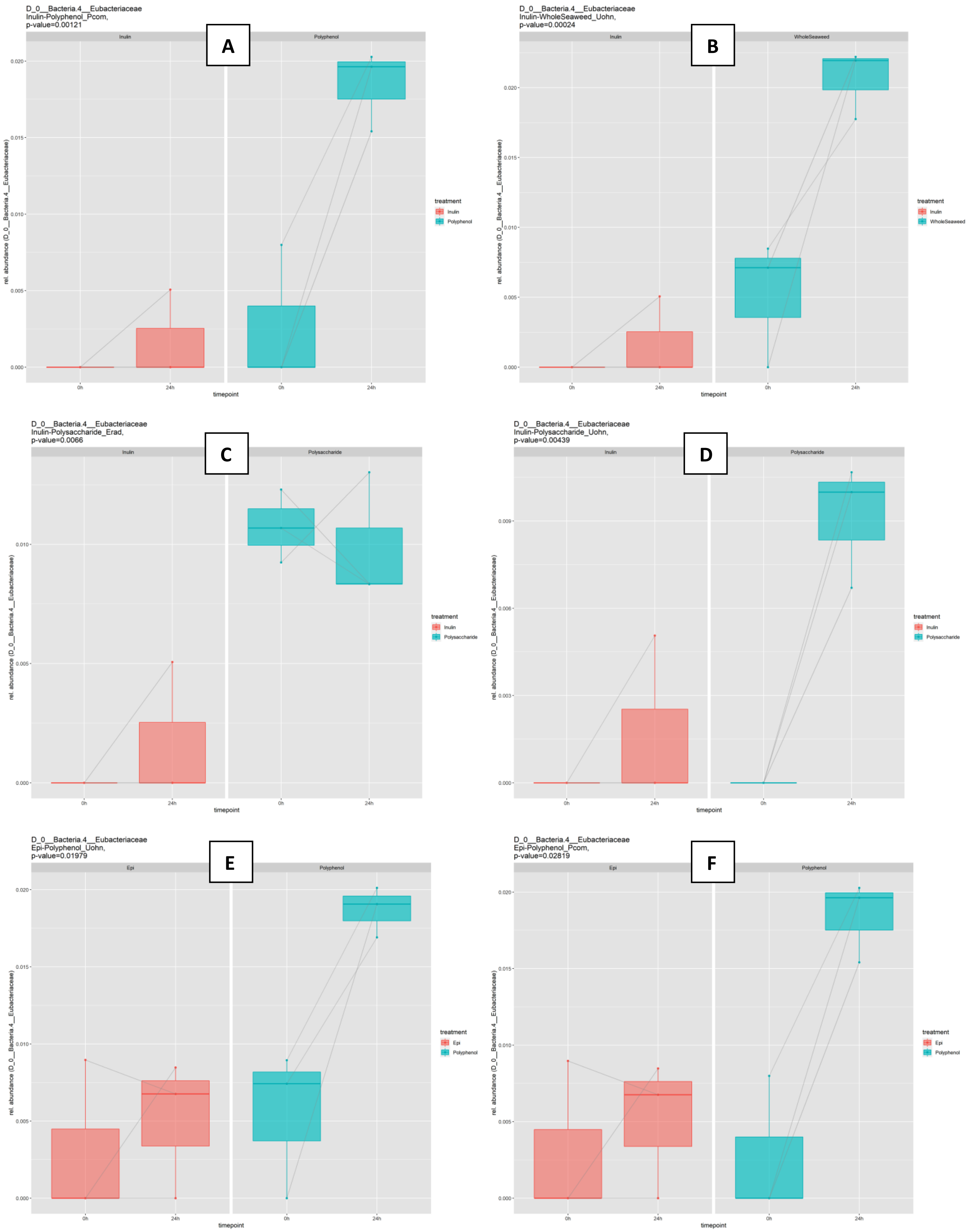


Figure S12. The abundance of the family Eubacteriaceae was enhanced by all WH, PS, and PP extracts compared to INU and EGCG (with the exception of *E. radiata* PP *vs* EGCG where there was no significant increase). Examples shown are (A) *P. comosa* PP (P = 0.00121), (B) *U. ohnoi* WH (P = 0.00024), (C) *E. radiata* PS (P = 0.0066) and (D) *U. ohnoi* PS (P = 0.000439) *vs* INU. (E) *U. ohnoi* PP (P = 0.01979) and (F) *P. comosa* PP (P = 0.02819) *vs* EGCG

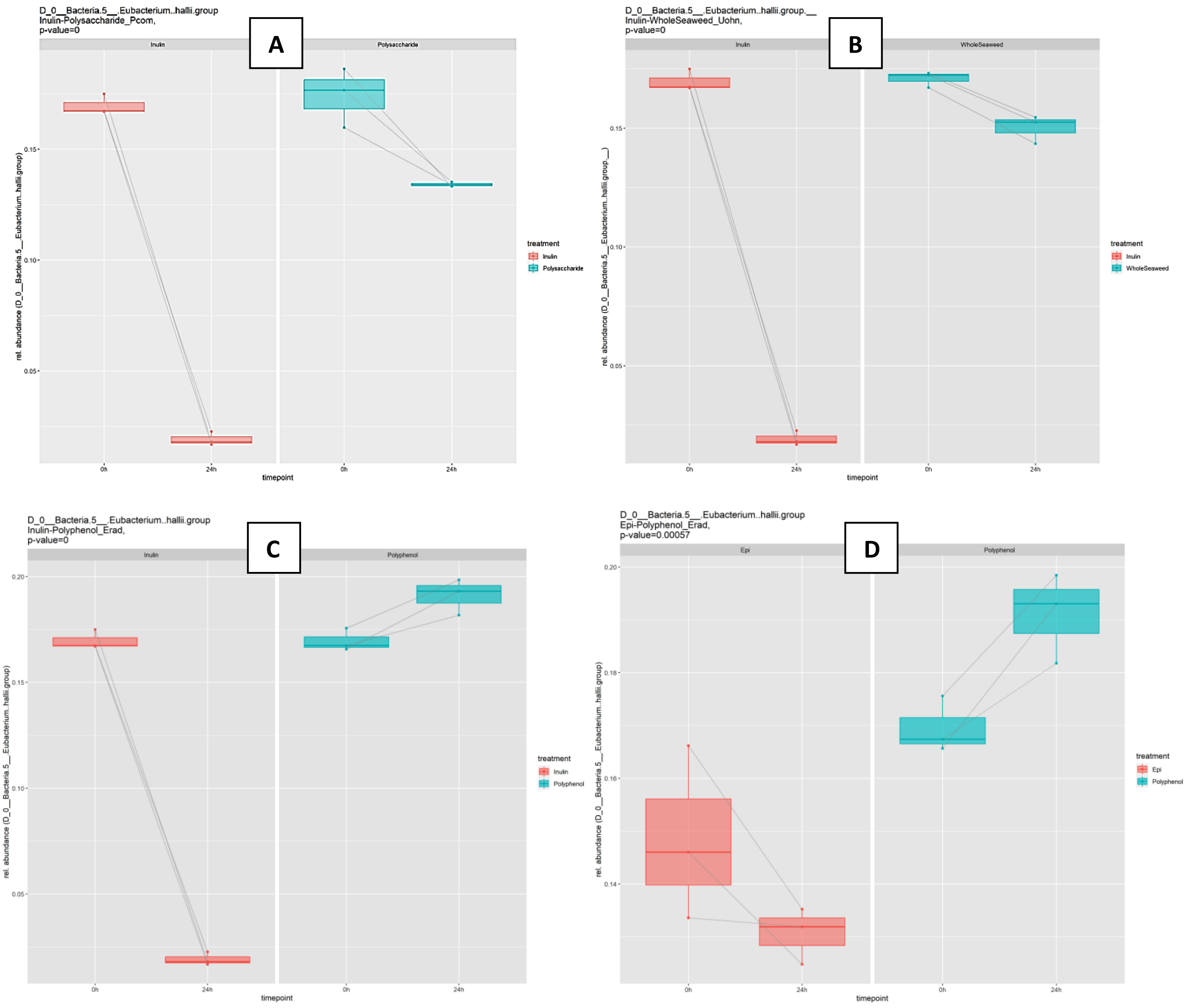


Figure S13. The abundance of the species *Eubacterium halii* was enhanced significantly by all WH, PS, and PP extracts compared to INU. Only *E. radiata* PP increased abundance compared to EGCG. Examples shown are (A) *P. comosa* PS (P = 0), (B) *U. ohnoi* WH (P = 0) and (C) *E. radiata* PP (P = 0) *vs* INU. (D) *E. radiata* PP (P = 0.00057) was the only polyphenol extract that enhanced abundance more so than the EGCG control.

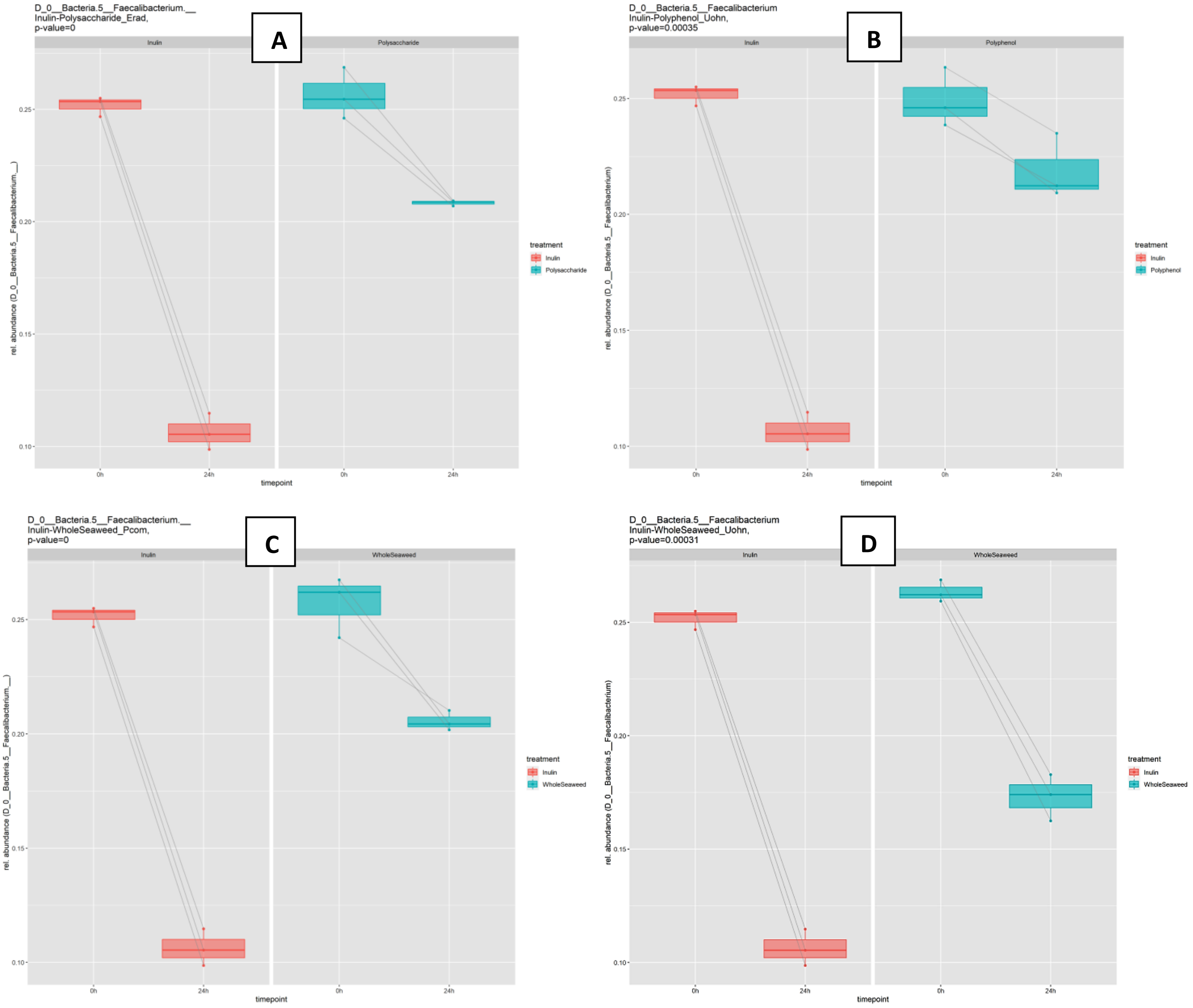


Figure S14. Genus Faecalibacterium increased significantly compared to INU in all WH, PS and PP extracts. Examples of enhancement shown are (A) *E. radiata* PS (P = 0), (B) *U. ohnoi* PP (P = 0.00035), (C) *P. comosa* WH (P = 0) and (D) *U. ohnoi* WH (P = 0.00031) *vs* INU

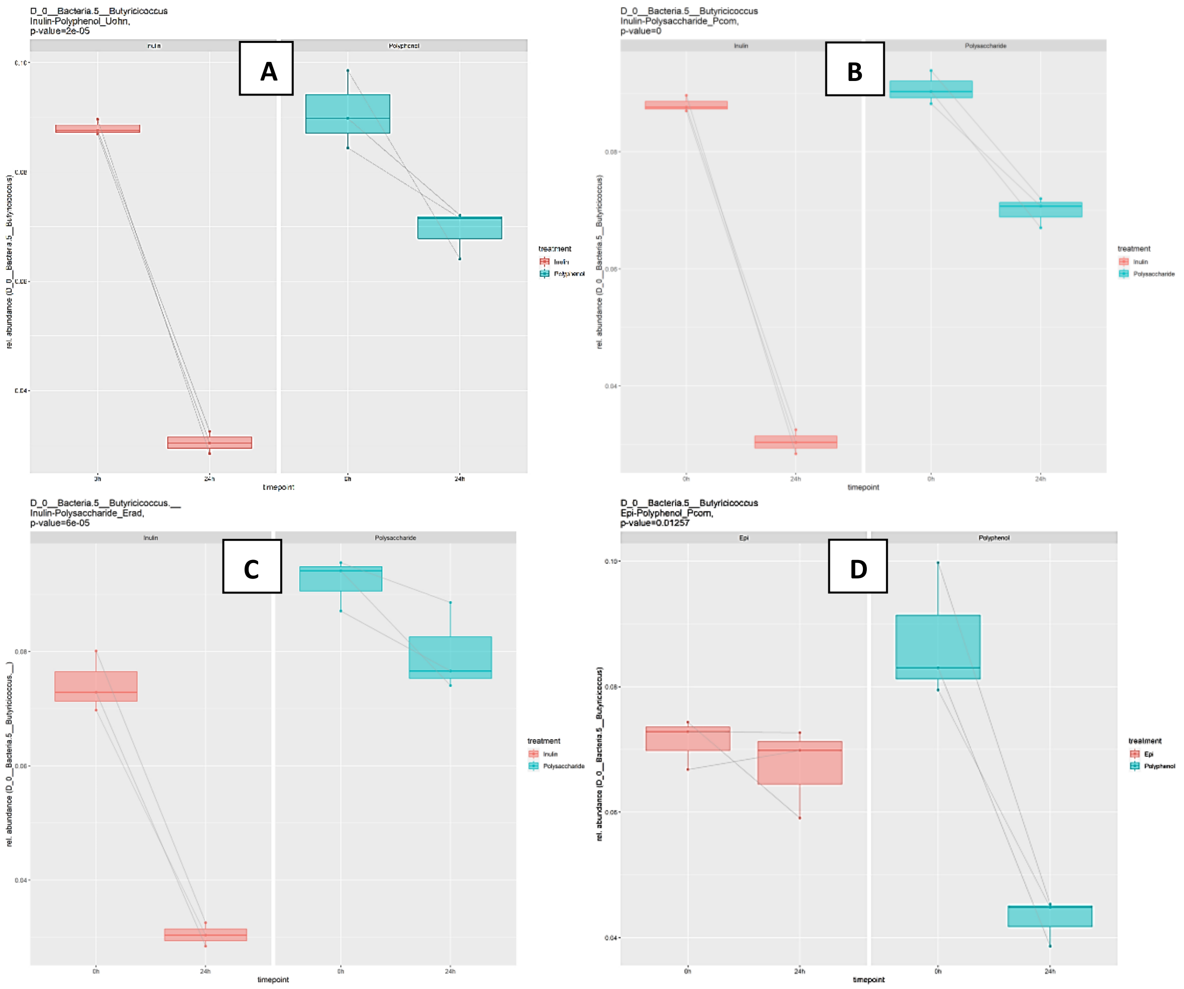


Figure S15. Compared to INU, genus Butyricicoccus was enhanced by all WH and PS ferments, but decreased compared to EGCG. Examples of enhancement shown are (A) *U. ohnoi* PP (P = 0.00002), (B) *P. comosa* PS (P = 0) and (C) *E. radiata* PS (P = 0.00006) *vs* INU, and (D) reduction by *P. comosa* PP *vs* EGCG (P = 0.01257)

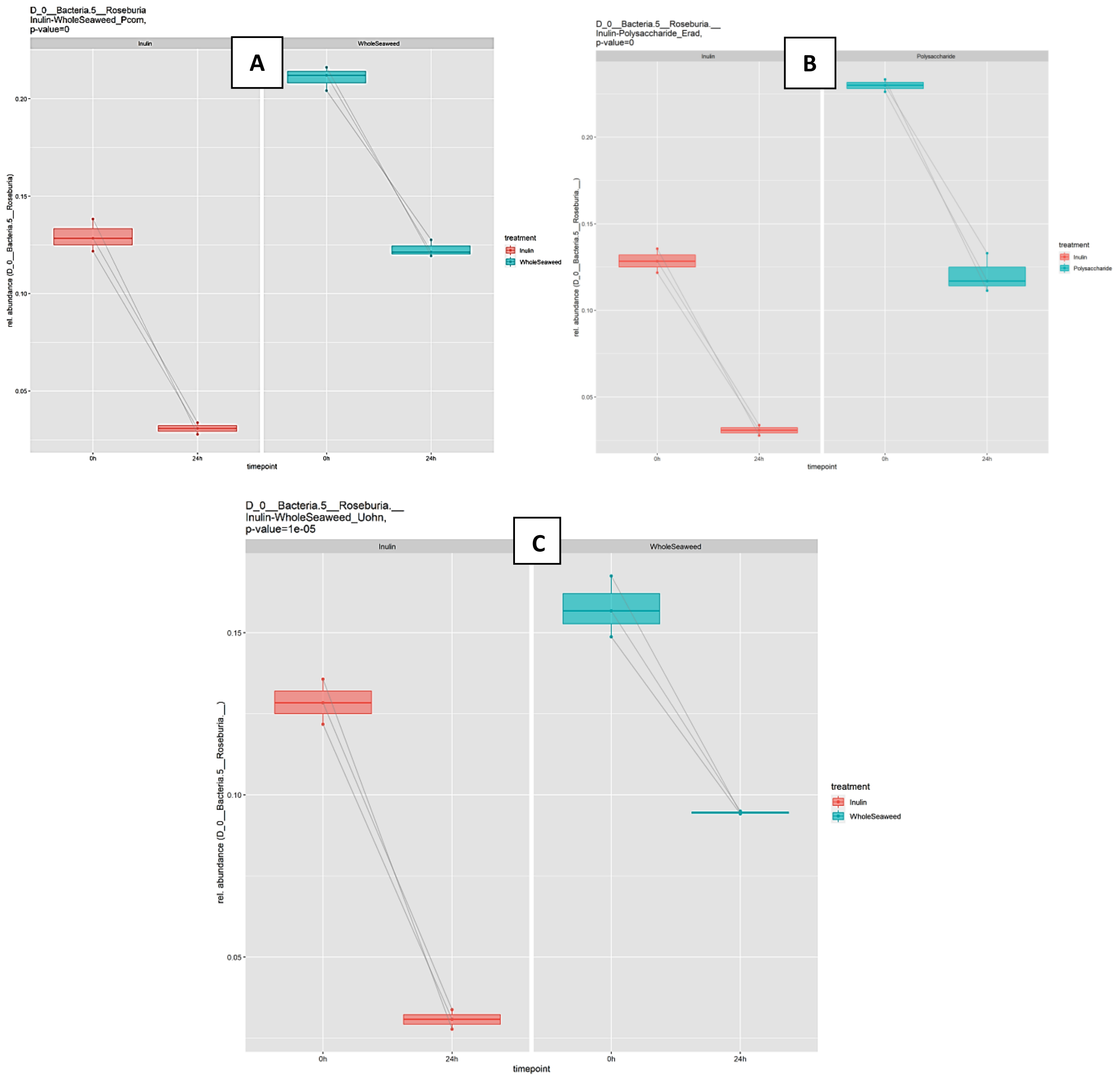


Figure S16. Genus Roseburia was enhanced by all WH and PS ferments but not by PP. Examples shown are (A) *P. comosa* PS (P = 0), (B) *E. radiata* PS (P = 0) and (C) *U. ohnoi* WH (P = 0.00001) *vs* INU

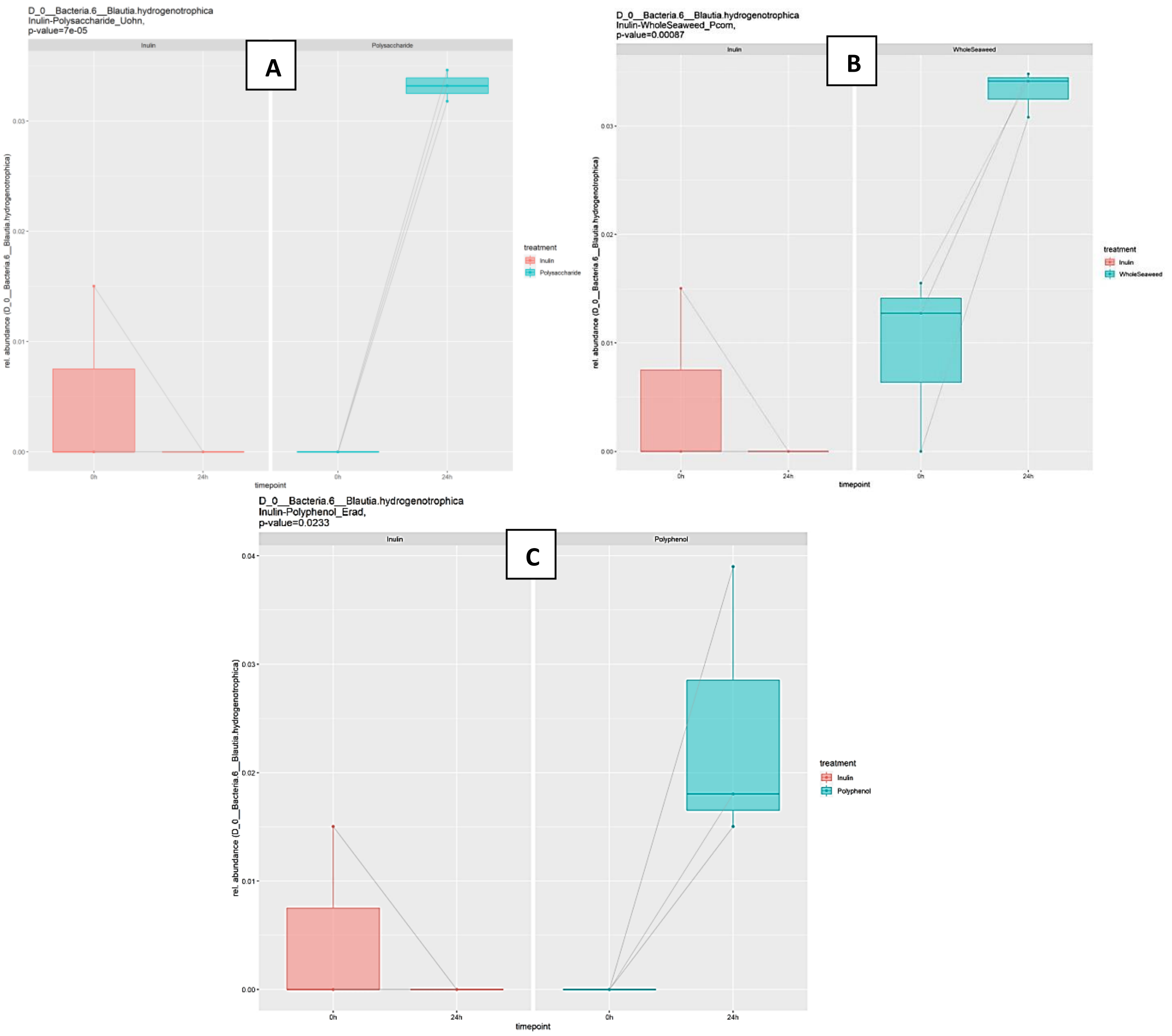


Figure S17. (A) *U. ohnoi* PS (P = 0.00007), (B) *P. comosa* WH (P = 0.00087) and (C) *E. radiata* PP (P = 0.0233) enhanced *Blautia hydrogenotrophica* abundance compared to INU

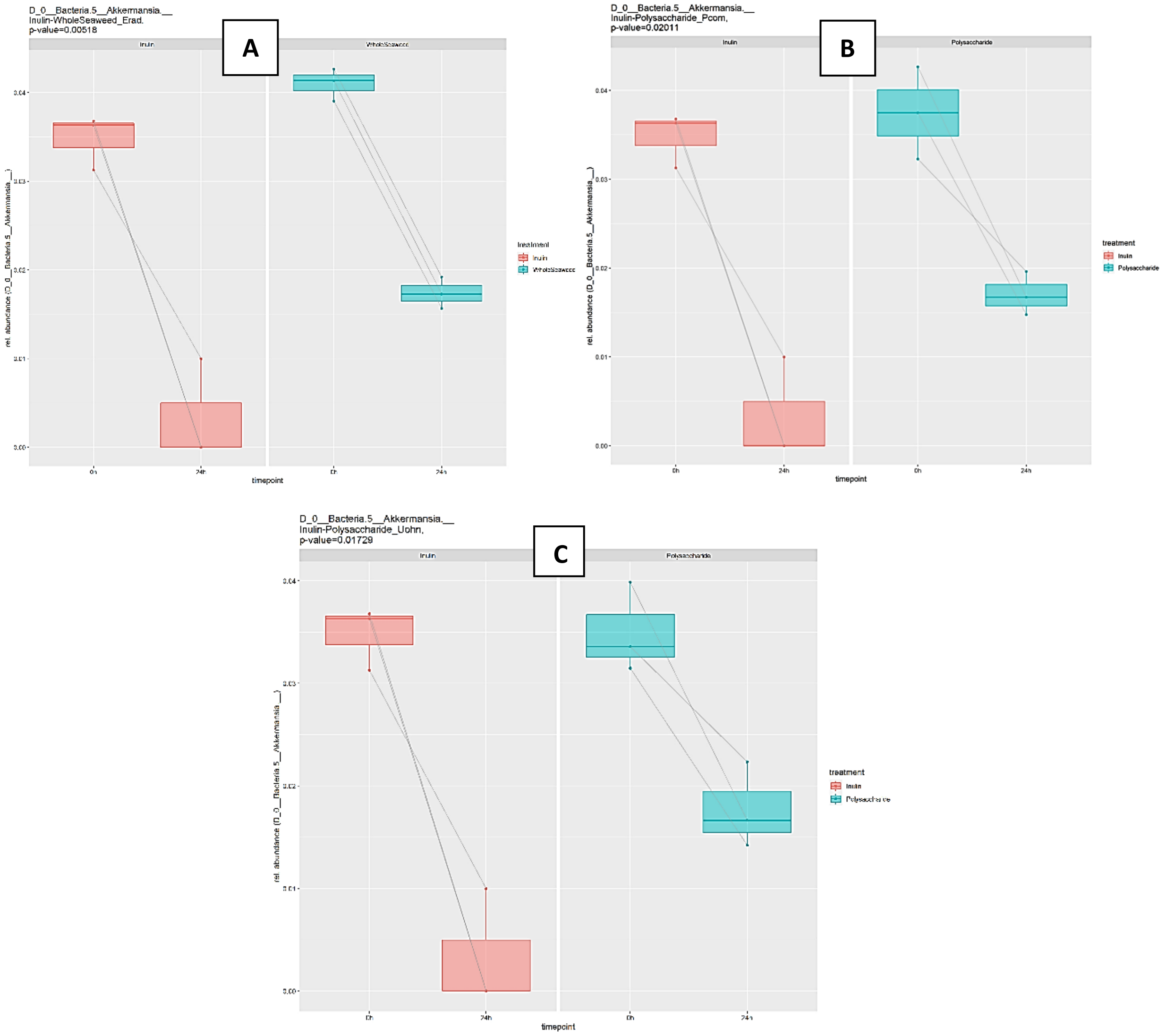


Figure S18. Genus Akkermansia was enhanced by all WH and PS ferments but not by PP. Examples shown are (A) *E. radiata* WH (0.00518), (B) *P. comosa* PS (P = 0.02011) and (C) *U. ohnoi* PS *vs* INU

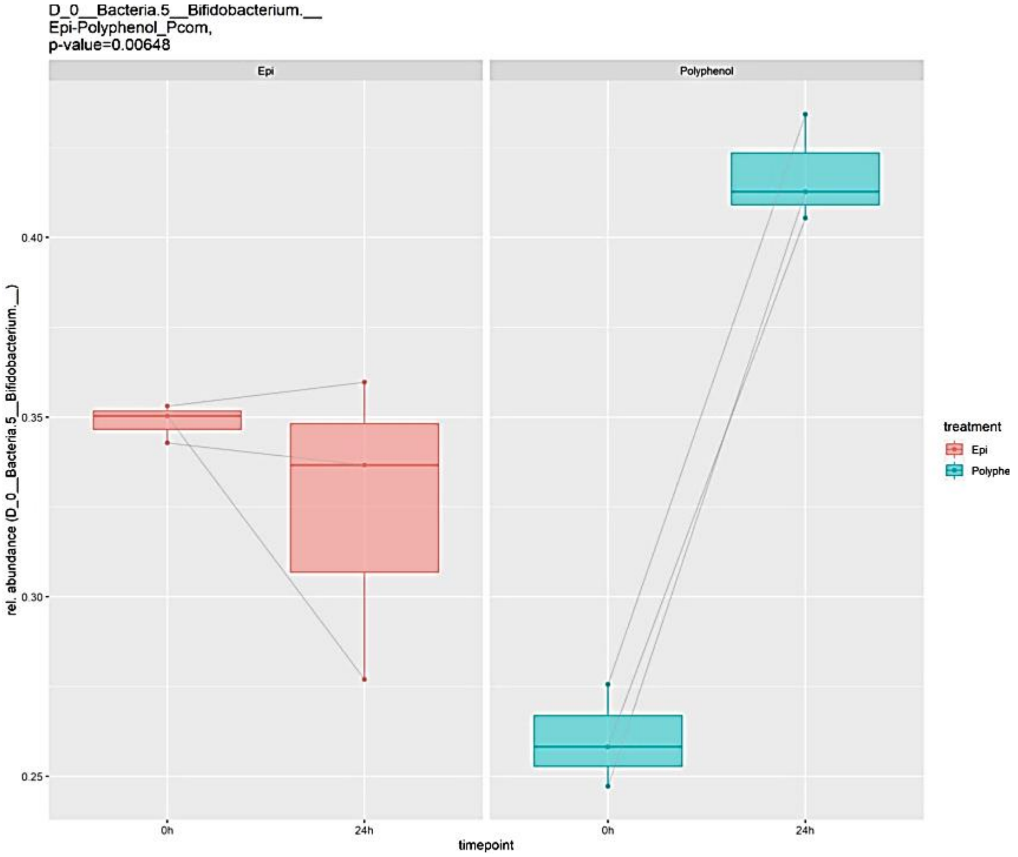


Figure S19. *P. comosa* PP was the only seaweed substrate to enhance Bifidobacteria compared to the EGCG control (P = 0.00648)

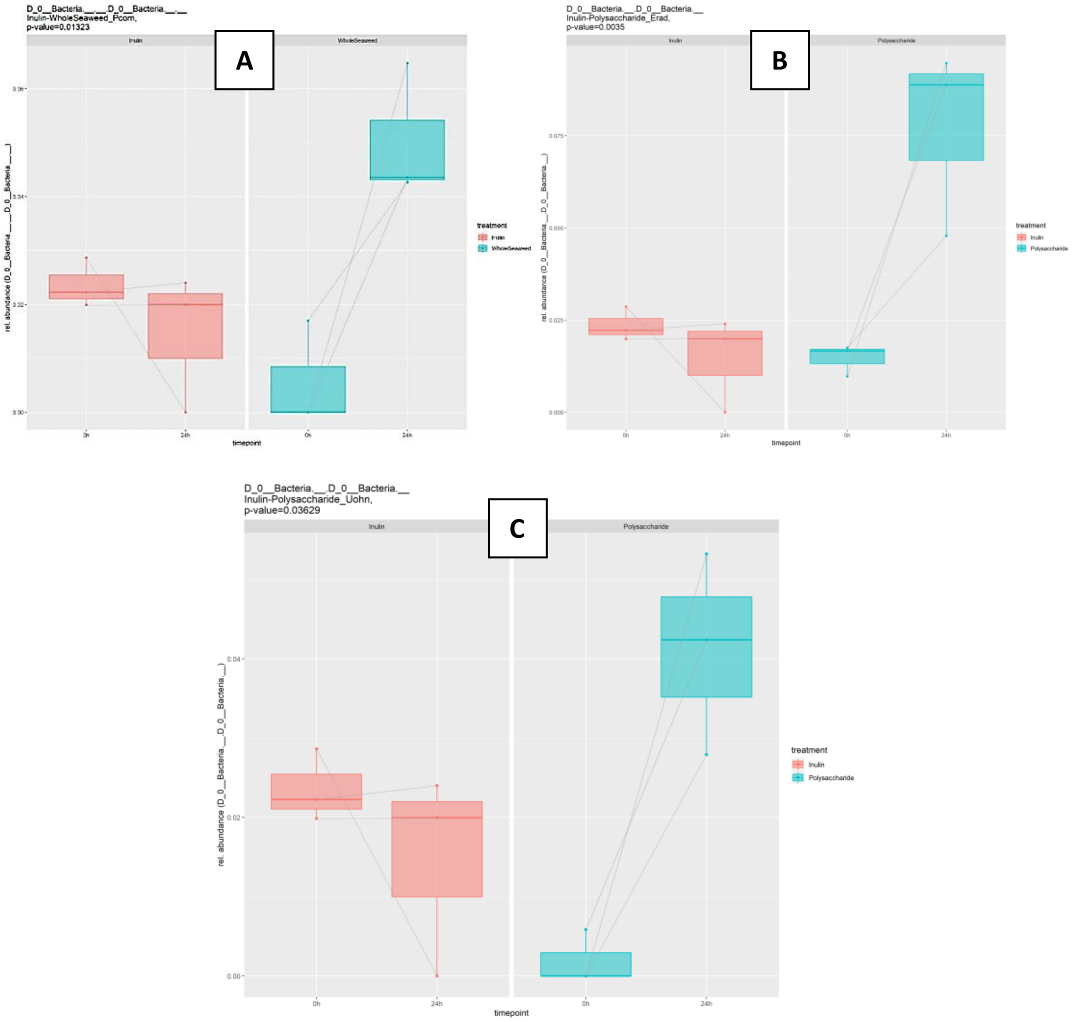


Figure S20. All PS and WH seaweed substrates increased the abundance of the phylum of unclassified 'Bacteria' *vs* INU suggesting that species richness was enhanced. Examples shown are (A) *P. comosa* WH (P = 0.01323), (B) *E. radiata* PS (P = 0.0035) and (C) *U. ohnoi* PS (P = 0.03629)

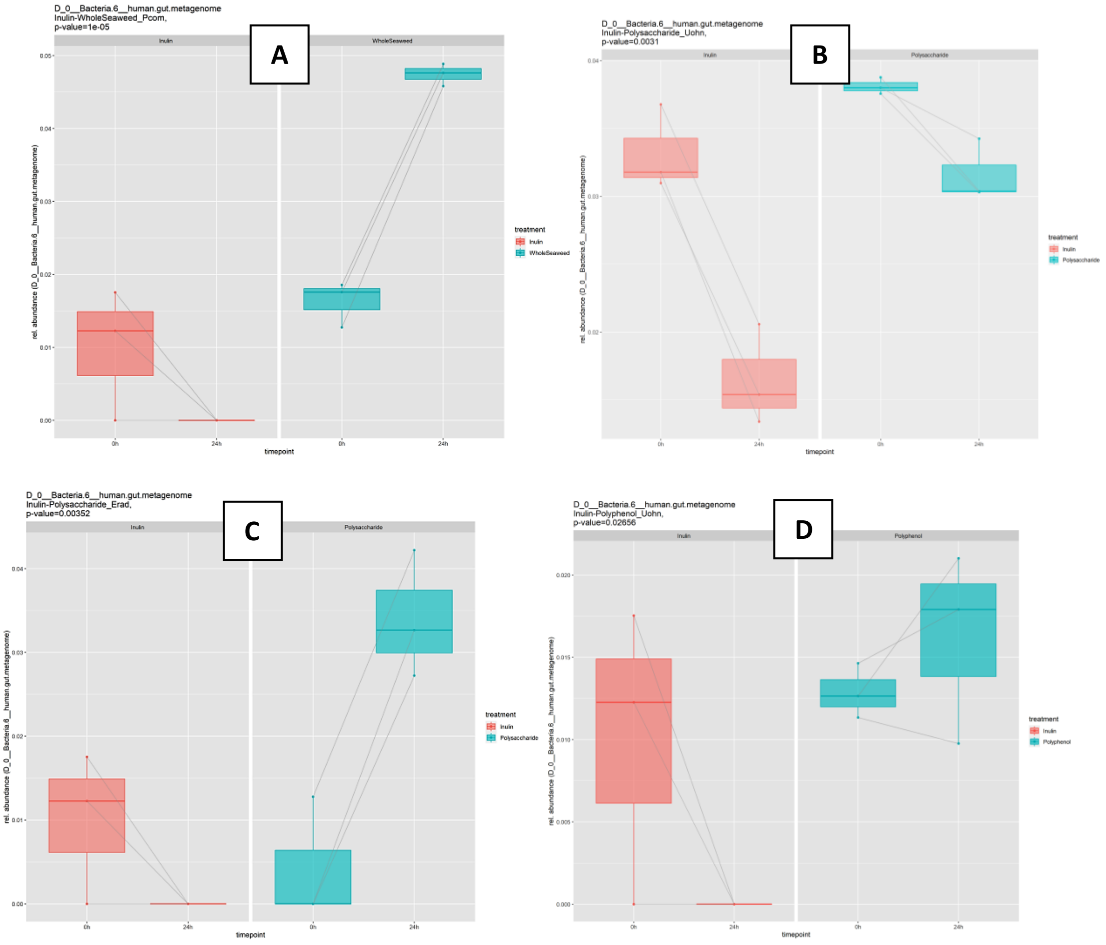


Figure S21. Human gut metagenome abundance was enhanced by all nine seaweed ferments *vs* INU. Examples shown are (A) *P. comosa* WH (P = 0.00001), (B) *U. ohnoi* PS (P = 0.0031), (C) *E. radiata* PS (P = 0.00352) and (D) *U. ohnoi* PP (P = 0.02656)

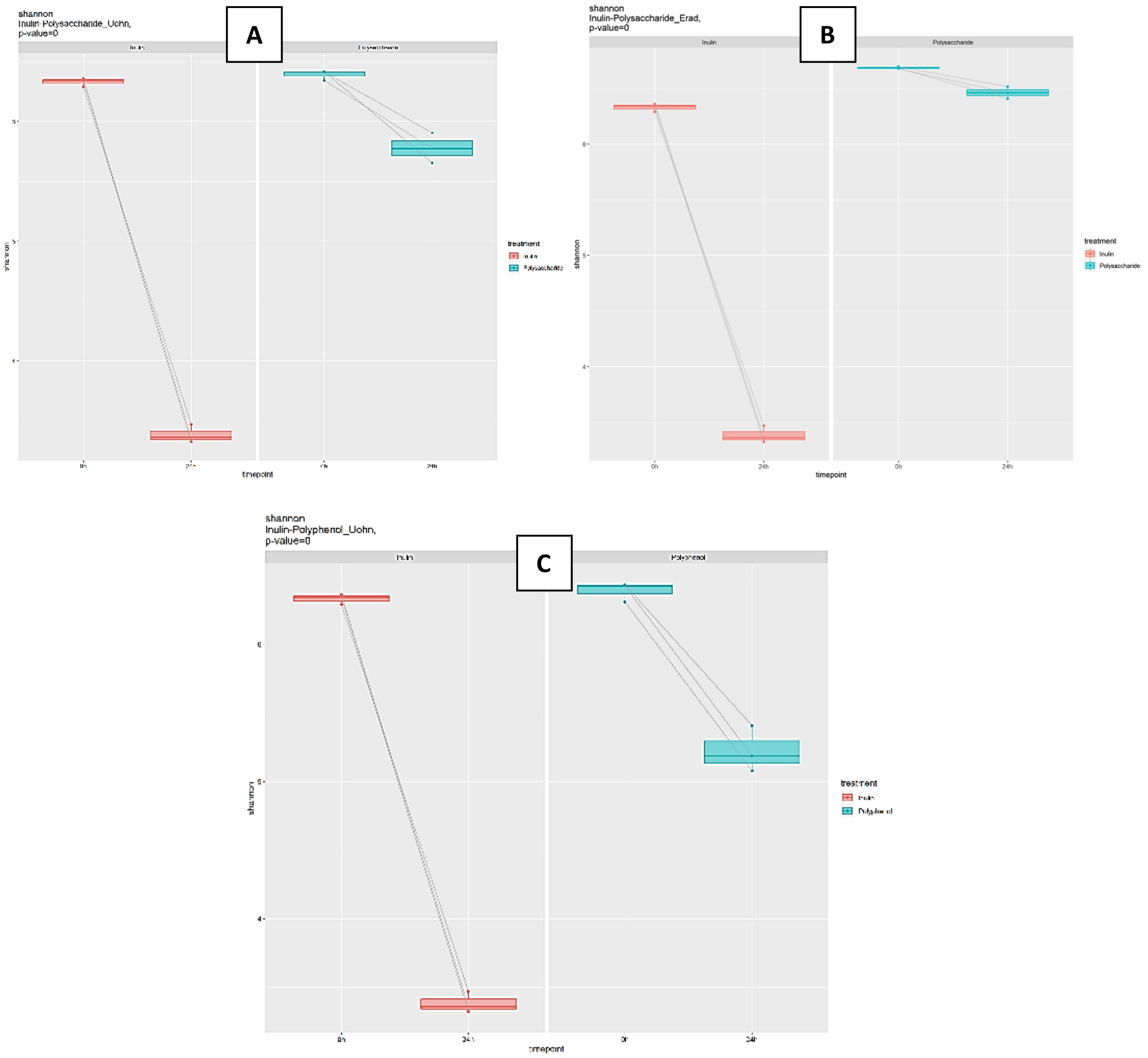


Figure S22. Shannon diversity index increased in all seaweed ferments *vs* INU. Examples shown are (A) *U. ohnoi* PS *vs* INU (P = 0), (B) *E. radiata* PS *vs* INU (C) and *U. ohnoi* PP *vs* INU (P = 0)

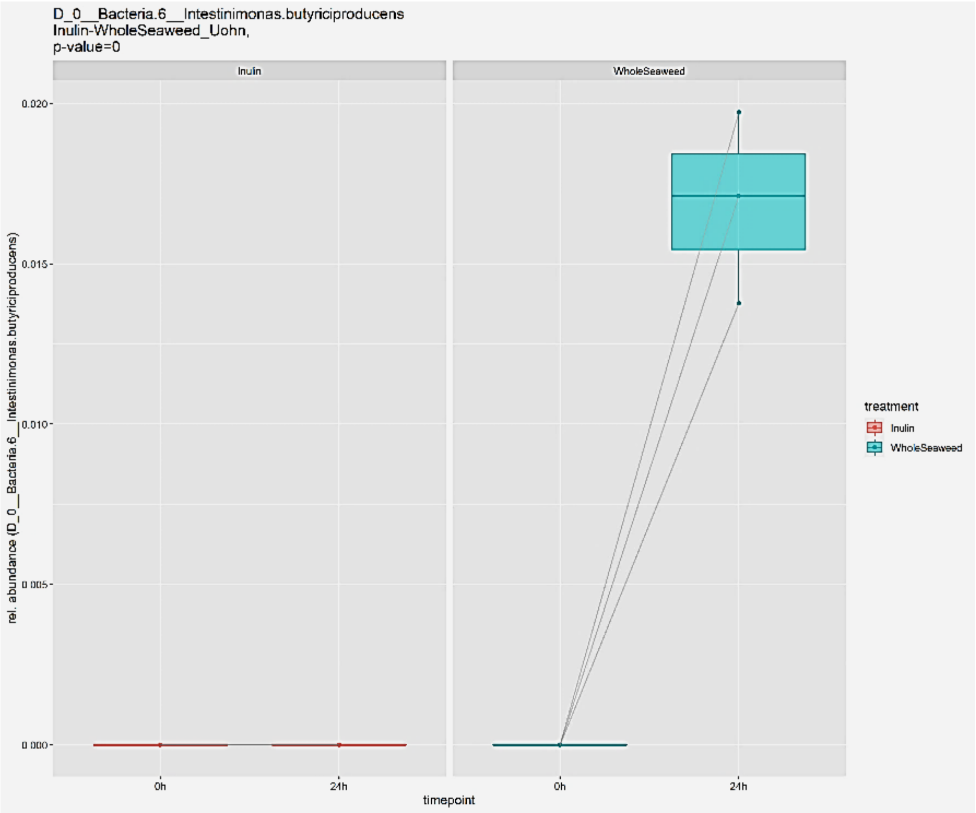


Figure S23. *Intestinimonas butyriciproducens* of the phylum Firmicutes was enhanced by *U. ohnoi* WH *vs* INU (P = 0)

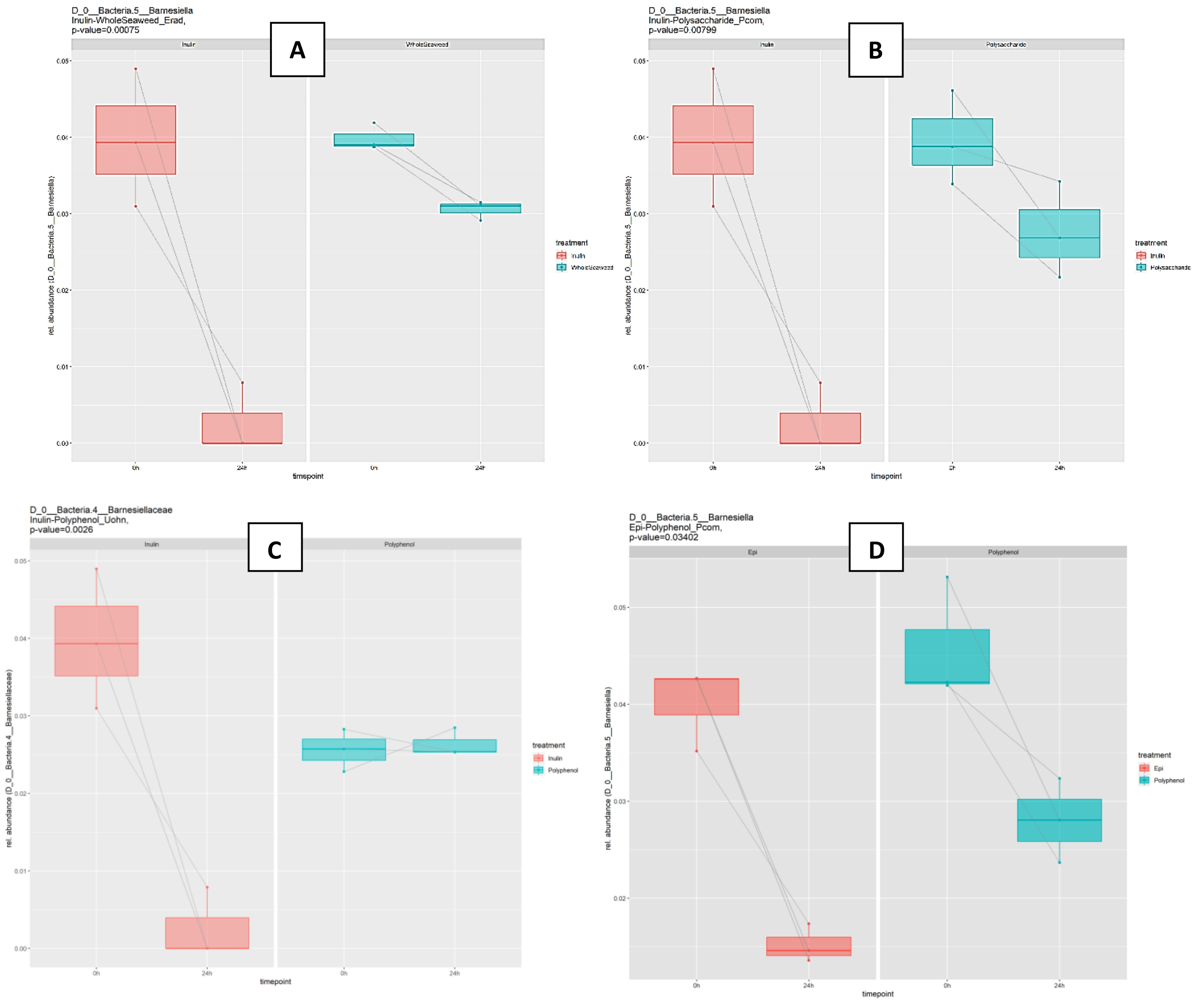


Figure S24. Genus Barnesiella abundance was enhanced in all WH, PS and PP ferments compared to INU and EGCG. Shown here are (A) *E. radiata* WH (P = 0.00075), (B) *P. comosa* PS (P = 0.00799) and (C) *U. ohnoi* PP (P = 0.0026) *vs* INU, and (D) *P. comosa* PP (P = 0.03402) *vs* EGCG

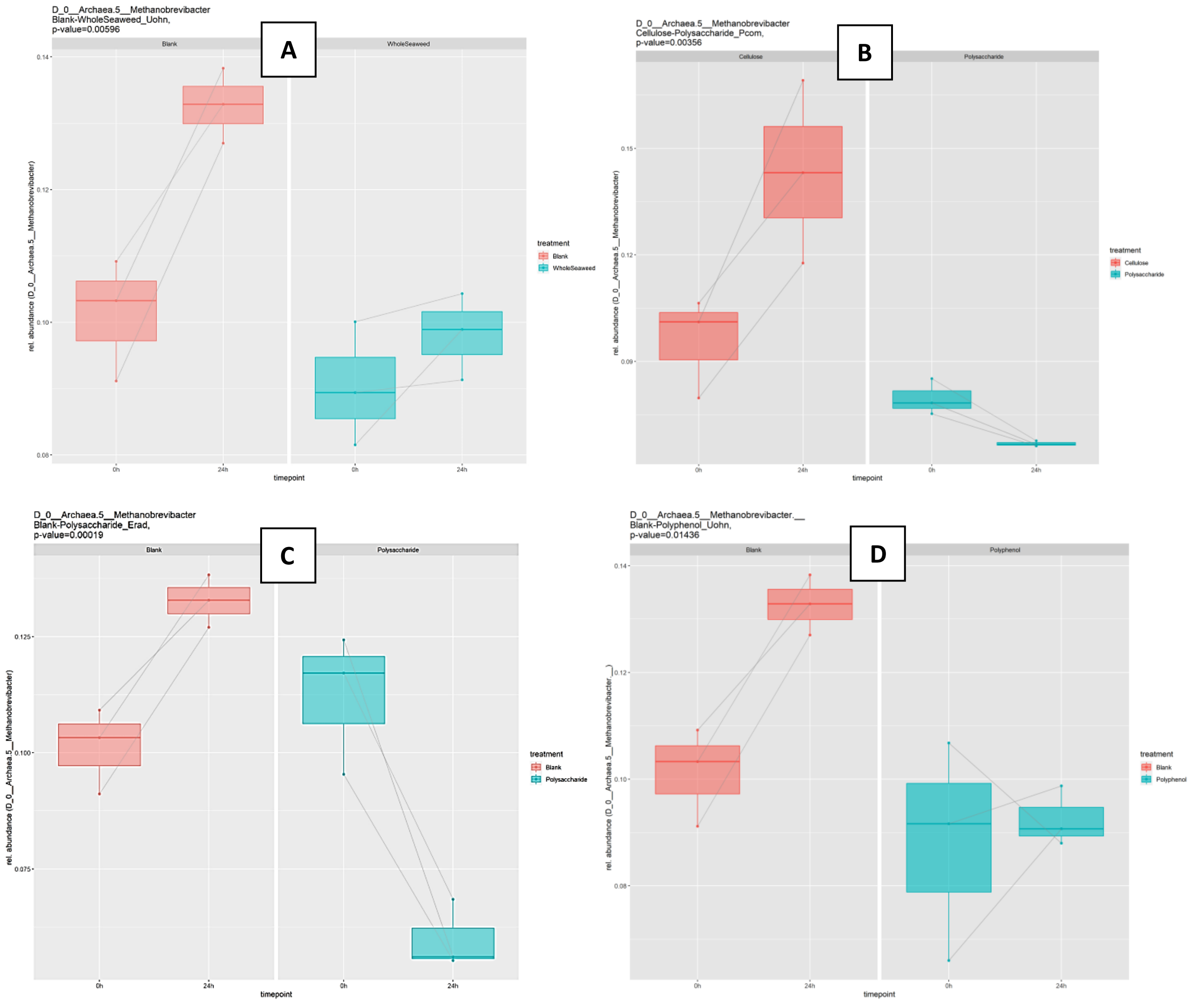


Figure S25. The abundance of Methanobrevibacter was reduced by all nine seaweed ferments *vs* the blank and cellulose. Examples shown are (A) *U. ohnoi* WH *vs* blank (P = 0.00596), (B) *P. comosa* PS *vs* cellulose (P = 0.00356) (C) *E. radiata* PS *vs* blank (P = 0.00019) and (D) *U. ohnoi* PS (P = 0.01436)
